# Single-Cell Transcriptomics of Immune Cells Reveal Diversity and Exhaustion Signatures in Non-Small-Cell Lung Cancer

**DOI:** 10.3389/fimmu.2022.854724

**Published:** 2022-07-06

**Authors:** Ying Zhao, Qilin Zhang, Kailin Tu, Yanmei Chen, Yuxuan Peng, Yinyun Ni, Guonian Zhu, Cheng Cheng, Yangqian Li, Xue Xiao, Chunyan Yu, Keying Lu, Yaxin Chen, Chengpin Li, Jun Tang, Gang Wang, Wenxin Luo, Wengeng Zhang, Guowei Che, Weimin Li, Zhoufeng Wang, Dan Xie

**Affiliations:** ^1^ Institute of Respiratory Health, Frontiers Science Center for Disease-related Molecular Network, Precision Medicine Key Laboratory of Sichuan Province, West China Hospital, Sichuan University, Chengdu, China; ^2^ Laboratory of Omics Technology and Bioinformatics, State Key Laboratory of Biotherapy, West China Hospital, Sichuan University, Chengdu, China; ^3^ Health Management Center, West China Tianfu Hospital, Sichuan University, Chengdu, China; ^4^ Department of Respiratory and Critical Care Medicine, Frontiers Science Center for Disease-related Molecular Network, West China Hospital, Sichuan University, Chengdu, China; ^5^ Department of Thoracic Surgery, West China Hospital, Sichuan University, Chengdu, China

**Keywords:** non-small cell lung cancer, tumor microenvironment, single-cell transcriptomic sequencing, CD8^+^ T cells, tumor-associated macrophages, regulatory B cells

## Abstract

Understanding immune cell phenotypes in the tumor microenvironment (TME) is essential for explaining and predicting progression of non-small cell lung cancer (NSCLC) and its response to immunotherapy. Here we describe the single-cell transcriptomics of CD45^+^ immune cells from tumors, normal tissues and blood of NSCLC patients. We identified three clusters of immune cells exerting immunosuppressive effects: CD8^+^ T cells with exhausted phenotype, tumor-associated macrophages (TAMs) with a pro-inflammatory M2 phenotype, and regulatory B cells (B regs) with tumor-promoting characteristics. We identified genes that may be mediating T cell phenotypes, including the transcription factors ONECUT2 and ETV4 in exhausted CD8^+^ T cells, TIGIT and CTL4 high expression in regulatory T cells. Our results highlight the heterogeneity of CD45^+^ immune cells in the TME and provide testable hypotheses about the cell types and genes that define the TME.

## Introduction

Immunotherapy can substantially improve the prognosis of patients with non-small-cell lung cancer (NSCLC) by inducing an effective adaptive antitumor immune response (1). The efficacy of such therapy depends strongly on the distribution and activity of immune cells within the tumor microenvironment (TME) and adjacent normal tissue (2). Thus, understanding the identities, phenotypes and activities of the various types of immune cells in the TME is essential for optimizing immunotherapy.

The cellular components of the TME are highly complex, with diverse populations of myeloid cells and lymphocytes playing important roles in inflammation, cancer immune evasion, and responses to immunotherapy treatment (3). The presence of myeloid cells in the TME is often linked to altered patient survival (4). For example, certain B cells can regulate T cells and TAMs to promote cancer progression (5). Few studies, however, have exploited this approach for identifying “exhaustion signatures” of immune cells that are associated with weak antitumor activity and therefore greater risk of progression and metastasis. Single-cell RNA sequencing (scRNA-seq), which can characterize cell diversity within the TME (6, 7), has shown many types of tumors to contain heterogeneous TAMs, dendritic cells (DCs) and other types of tumor-infiltrating myeloid cells (8). We have previously detected a range of immune cell types in lung adenocarcinoma tissue using scRNA-seq (9), but the global immune landscape is still unknown.

Here we applied scRNA-seq to characterize the diversity of immune cells within NSCLC tissue, normal tissue and blood. We found a much broader range of lymphocytes and myeloid cells within tumors than normal tissue. Their phenotypic heterogeneity was associated with diverse gene expression patterns, suggesting that the TME comprise diversity states of exhaustion or activation, instead of only a few discrete states. Our findings will help guide future studies of NSCLC pathogenesis and treatment.

## Materials and Methods

### Patients and Ethics Statement

Patients diagnosed with NSCLC at West China Hospital of Sichuan University in China between 2018 and 2020 were prospectively enrolled in this study. All patients were treated surgically, and none received neoadjuvant therapy before surgery. Tumors and matched distal normal lung tissues were obtained during surgery. Normal tissues were obtained 5cm apart from tumor margin. All samples were evaluated by two pathologists to determine the pathological diagnosis and tumor cellularity. Cancer was staged according to the TNM system of the American Joint Committee on Cancer (8th edition).

### Sample Preparation

Freshly resected lung tissue was procured intraoperatively from patients undergoing lobectomy for focal lung tumors. Normal lung tissues were obtained from uninvolved regions, and the lung lobe and location along the airway or periphery were recorded. Tissues were mechanically and enzymatically dissociated using a tumor dissociation kit (Miltenyi Biotec, Bergisch Gladbach, Germany). Suspensions were filtered through a 40-μm cell strainer to yield single-cell suspensions.

Prior to surgery, peripheral blood (3ml) was collected in EDTA tubes and isolated using HISTOPAQUE-1077 (Sigma-Aldrich, BA) according to the manufacturer’s instructions. After centrifugation, lymphocyte cells at the interface between plasma and HISTOPAQUE were carefully transferred to a new tube, then washed twice with phosphate-buffered saline (PBS, Invitrogen). Lymphocyte cells were re-suspended in PBS supplemented with 1% fetal bovine serum (FBS, ScienCell).

Cells were blocked in Hanks buffered saline solution containing 3% FBS, and then stained first with CD45-Vioblue direct conjugate antibody (catalog no. 130-092-880, Miltenyi Biotec). Cells were washed with PBS, then stained with 7-aminoactinomycin D (Life Technologies, Carlsbad, CA, USA). CD45^+^ cells were sorted on a FACSAria Fusion (Becton Dickinson, Franklin Lakes, NJ, USA).

### Single-Cell RNA Sequencing

Single-cell suspensions were loaded onto a Chromium Single Cell Chip (10x Genomics) and co-encapsulated with barcoded Gel Beads according to the manufacturer’s instructions, at a target capture rate of ~5000 cells per sample. Captured mRNA was barcoded during cDNA synthesis, and the resulting cDNA was converted into pooled single-cell RNA sequence libraries. All samples from a given donor were processed simultaneously using the Chromium Controller (10x Genomics), and the resulting libraries were prepared in parallel in a single batch. The libraries were sequenced using NovaSeq 6000 system (Illumina) according to the manufacturer’s instructions. All libraries were sequenced with an 8-base index read, a 26-base read 1 containing cell-identifying barcodes and unique molecular identifiers (UMIs), and a 98-base read 2 containing transcript sequences.

### Single-Cell RNA Data Processing

Reads were aligned and the UMI matrix was generated using the Cell Ranger toolkit (version 3.0.2; 10X Genomics) and the reference genome GRCh37 (hg19). Then the gene expression matrices for all peripheral blood, tumor and normal samples were combined in R (version 3.6.1) and converted to a Seurat object using the Seurat package in R (version 3.2.2). Cells were removed if no more than 200 genes or if more than 6000 genes were found to be expressed, or if >10% of UMIs corresponded to the mitochondrial genome. The number of cells retained for each feature was calculated, and only genes that were expressed in at least five cells per feature were retained. We normalized the count matrix of remaining cells to TP10K using the “NormalizeData” function in the Seurat package.

We corrected for batch effects using the “FindIntegrationAnchors” function in the Seurat package as recommended: we scaled each dataset, selected 2000 HVGs as input to compute integration anchors, then integrated the batches using the anchors. Linear regression was used to log-normalize gene expression matrices to total cellular read-counts and mitochondrial read-counts using the “ScaleData” function.

### Identification of Major Immune Cell Types and Subtypes

We selected the 2000 most variably expressed genes to identify major cell types. To reduce dimensionality, variably expressed genes were summarized using principal component analysis, then the principal components were summarized using t-SNE dimensionality reduction (“RunTSNE function”). The number of principal components depended on the dataset. Data were clustered using the graph-based clustering approach in the “FindClusters” function of the Seurat package, with the resolution parameter of 0.5 and other parameters set to their defaults, and 70 cells were the lowest cell numbers per cluster. This method identifies cell clusters using shared nearest-neighbor modularity optimization-based clustering. We then classified clusters as the following cell types, based on average expression of the indicated curated gene sets: epithelial cells (EPCAM, KRT19, KRT18, KRT5, and KRT15), myeloid cells (LYZ, CD68, MS4A6A, CD1E, IL3RA, and LAMP3), T cells (CD2 and CD3D/E/G), NK cells (XCL1, KLRD1 and KLRF1) and B cells (CD79A/B, CD19, and MS4A1). Not all clusters were in the patients, but the proportions of immune cell types are stable.

We further clustered T/NK cells, B cells, myeloid cells individually. We used the paran function in the R package paran to calculate the number of PCs (iterations = 100, centile = 50). To reduce dimensionality, the principal components were summarized using RunUMAP, RunTSNE function in the R package Seruat (reduction = ‘pca’, dims=1: retain_PC). After that, we use FindNeighbors (reduction = ‘pca’, dims=1:retain_PC) and FindClusters(resolution = 0.5) from the R package Seruat to perform reclustering. Within T lineage, we used the following markers for subtype identification: CD8^+^ exhausted T (CD8A, LAG3, and TIGIT), CD8^+^ cytotoxic T (CD8A, GZMA, GZMK, GZMB, GZMH), CD4^+^ naïve T (CCR7, LEF1, IL7R, and SELL), CD4^+^ Tregs (FOXP3, IL2RA, and IKZF2). KLRC1, KLRD1, and NKG7 were used as the markers of NK cells. Similarly, we distinguished follicular B cells (MS4A1, CD19, CD79A, CD79B) from plasma cells (CD38, MZB1, TNFRSF17, and SDC1) among the B cell lineage. Regulatory B cells (IL10, CD5, TLR4) and GC B cells (LMO2, AICDA, RGS13, GCSAM) were clustered in the B cell lineage. For the myeloid clusters, macrophages were positive for canonical marker CD68 and CD163, and alveolar mac markers MARCO and FABP4. Other myeloid cell types were confirmed by specific marker genes including classical monocytes (LYZ, VCAN), DC2 (FCER1A, CD1C), and DC3(CCR7, CLEC4C).

### Identification of the Marker Gene Signatures of Immune Cell Subclusters

To identify marker gene signatures for each of the 32 immune subclusters within myeloid, T/NK and B cell types, the “FindMarkers” function in the Seurat package was used to compare cells within the given subcluster Marker genes for a subcluster were defined as genes (a) whose expression was detectable in >25% of the cells in the given subcluster, and (b) whose average expression was at least 1-fold higher in the given subcluster than in the other subclusters of the same cell type. Based on these parameters, we were able to identify marker genes for 25 of 32 subclusters. The marker genes for each subcluster make up the gene expression signature for the cell type.

To determine whether a given cell type was enriched in one tissue relative to other tissues, we calculated the ratio of observed cell number to expected cell number (R_o/e_) for each cluster, where the expected cell number was determined from the chi-squared test. We assumed that a cluster was enriched in a tissue if R_o/e_ > 1.

To identify genes differentially expressed between normal lung samples and matched tumor samples, the “FindMarkers” function in the Seurat package was applied to each subcluster. A differentially expressed gene associated with p < 0.05 was considered to be a tissue-specific gene. We then calculated the Z-score using the “p.to.Z” function in the NCmisc package in R (version 1.1.6).

### Survival Analysis

The potential influence of genes or gene sets derived from specific cell clusters on patient survival was explored using the lung adenocarcinoma (LUAD) dataset on gene expression in The Cancer Genome Atlas (http://xena.ucsc.edu/) and clinical data in the Genomic Data Commons Data Portal (https://gdc-portal.nci.nih.gov/). Patient cohorts were stratified into high- or low-expression groups relative to the median expression level. After correcting clinical covariates (age, sex, tumor stage) using Cox proportional hazard modeling in the survival package in R, we plotted Kaplan-Meier survival curves using the ggsurvplot function in R. Differences were assessed for significance based on p values from the Cox regression models in the survival package.

### Trajectory Analysis

Trajectory analysis was performed separately for the CD8^+^ T cells and CD4^+^ T cells using Slingshot (version 1.4.0). For CD8^+^ T cells, we selected cluster 10 as the starting cluster (“start.clus = 10”). The global lineage structure was identified using a cluster-based minimum spanning tree, and principal curves were fit simultaneously to describe each lineage.

### Gene Set Variation Analysis

The hallmark and metabolic pathways in the Molecular Signature Database (10) were assessed for activation in individual cells using gene set variation analysis with the GSVA package (version 1.34.0).

### SCENIC Analysis

We used the pySCENIC package (version 0.10.2), a lightning-fast Python implementation of the “single-cell regulatory network inference and clustering” (SCENIC) pipeline (11). The search space around the transcriptional start site was determined using the following six gene-motif rankings: hg19-500bp-upstream-10species, hg19-tss-centered-5kb-10species, hg19-tss-centered-10kb-7species, hg19-tss-centered-5kb-7species, hg19-500bp-upstream-7species, and hg19-tss-centered-10kb-10species. The 20-thousand motif database was used for RcisTarget and GENIE3.

### Culture and Transfection of CD8^+^ T Cells

CD8^+^ T cells were isolated from blood of the NSCLC patients. The blood was diluted 1:1 with PBS, and PBMC were isolated on a Ficoll-Paque Premium gradient (GE) according to the manufacturer’s instructions. Human CD8^+^ T cells were then isolated from the PBMC using CD8 microBeads, and cultured with human CD3/CD28 T cell activator beads (StemCell) and 100 U/mL recombinant human IL-2 (Miltenyi Biotec, Bergisch Gladbach, Germany) in X-VIVOTM 15 medium supplemented with 10% FBS and 1% penicillin/streptomycin at 37°C in a humidified incubator with 5% CO_2_. After 14 days of incubation, CD8^+^ T cells (1x10^6^) were electroporated with 10 μg of overexpression plasmid encoding SOX2, ONECUT2 or ETV4 using a NEPA21 system (Nepa Gene). All experiments were performed with mycoplasma-free cells.

### Flow Cytometry

The CD8^+^ T cells were labeled with an APC-conjugated against CD8 (1:100 dilution) for 15 min on ice in the dark. Then, the cells were washed, fixed, and permeabilized using the Transcription Factor Buffer Set (BD, USA) according to the manufacturer’s instructions. The cells were then stained with PE-conjugated antibody against GZMB (1:50 dilution) and FITC-conjugated antibody against PRF1 (1:50 dilution). Flow cytometry was performed on a Moflo Astrios EQ system (Beckman Coulter) and FlowJo software.

### RNA Extraction and Quantitative Real-Time PCR

Total cell RNA was extracted using the RNeasy Mini Kit (Qiagen) and converted into cDNA using the iScript cDNA Synthesis Kit (Bio-rad). SYBR Green Supermix (Bio-rad) was used for real-time quantitative PCR. The reaction conditions and PCR system were operated in accordance with the instructions. All sequences were designed and synthesized by TSINGKE Chengdu, China) and listed in the Oligonucleotides table in Key Resources (GZMB forward, GGCTTCCTGATACGAGACGA; GZMB reverse, CTTGGCCTTTCTCTCCAGCT; PRF1 forward, ACCAGGACCAGTACAGCTTC; PRF1 reverse, GGGTGCCGTAGTTGGAGATA; β-actin forward, CCTTCCTGGGCATGGAGTC; β-actin reverse, TGATCTTCATTGTGCTGGGTG). Levels of target mRNAs were measured using the 2^-ΔΔCt^ method relative to the level of β-actin mRNA.

### Data Availability

The generated WES, WGS, and RNA-seq data in this study have been deposited to Genome Sequence Archive (GSA) in BIG Data Center, Beijing Institute of Genomics (BIG) under accession number HRAXXXX. The transcriptome data of TCGA LUAD were collected from the following web-links https://portal.gdc.cancer.gov/projects/TCGA-LUAD. The human-specific databases for RcisTarget were downloaded from (https://resources.aertslab.org/cistarget/databases/homo_sapiens/hg19/refseq_r45/mc9nr/gene_based/hg19-500bp-upstream-7species.mc9nr.feather) and (https://resources.aertslab.org/cistarget/databases/homo_sapiens/hg19/refseq_r45/mc9nr/gene_based/hg19-tsscentered-10kb-7species.mc9nr.feather).

## Results

### Landscape of Immune Cells in the NSCLC TME

**Figure 1:**
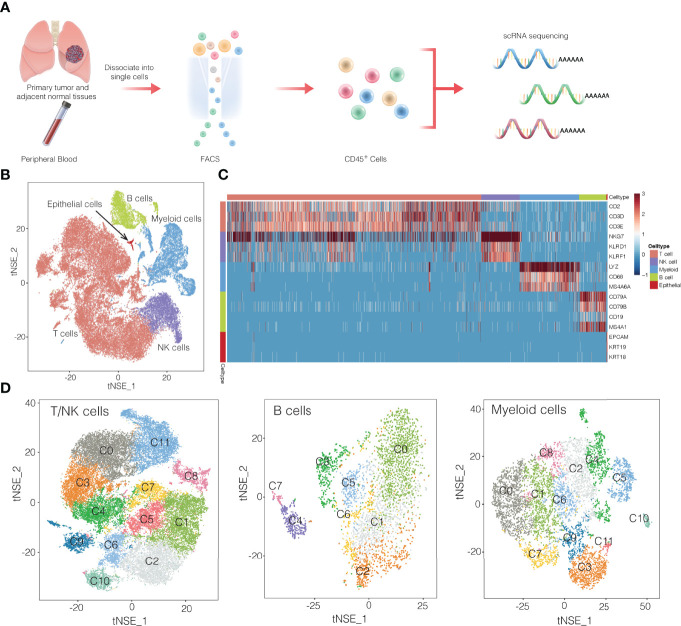
Overview of single-cell transcriptomic profiling of NSCLC samples. **(A)** Workflow showing the collection and processing of fresh samples from NSCLC for single-cell RNA sequencing (scRNA). FACS, fluorescence-activated cell sorting. **(B)** t-SNE plots of cells from the 16 samples profiled in this study. Different cell types were colored differently. **(C)** Heatmap of selected marker genes of 5 major cell types. Red: high expression; blue: low expression. **(D)** t-SNE projection of the expression profiles of the T cells/natural killer (NK) cells, myeloid cells, and B cells that passed quality control. Immune cell subsets, defined by 32 unique clusters, are annotated and marked by color code.

Using fluorescence-activated cell sorting, we collected CD45^+^ cells from tumors, normal tissues, and blood from 13 patients with NSCLC (**Figure 1A** and **Supplementary Figure 1** **)**. From these 16 samples (**Supplementary Table 1**), single-cell transcriptomes were determined using the droplet-based system of 10× Genomics Chromium(**Supplementary Table 2**). After filtering for data quality, about ~0.2 billion unique transcripts were obtained from 55,501 cells. We classified cells into transcriptomic clusters based on principal component analysis and a graphing method (12). Based on the average expression of curated gene sets, we annotated the clusters as myeloid cells, T cells, natural killer (NK) cells, B cells (**Figures 1B–D**). Notably, very few cells expressed epithelial markers, which may be due to the cell-cell interaction between epithelial cancer cells and immune cells (9). The gene expression data showed that each immune cell cluster contained cells from multiple patients (**Supplementary Figure 2**). Next, we identified 32 subclusters within the three major cell clusters of T/NK cells, myeloid cells, and B cells (**Figure 1D**). Subsequently we identified signature genes for each of the immune subclusters, successfully developing the immune subcluster signatures. (**Supplementary Figures 3, 4**, and **Supplementary Table 3**). These data may provide a useful reference atlas for studying NSCLC TME.

**Figure 2:**
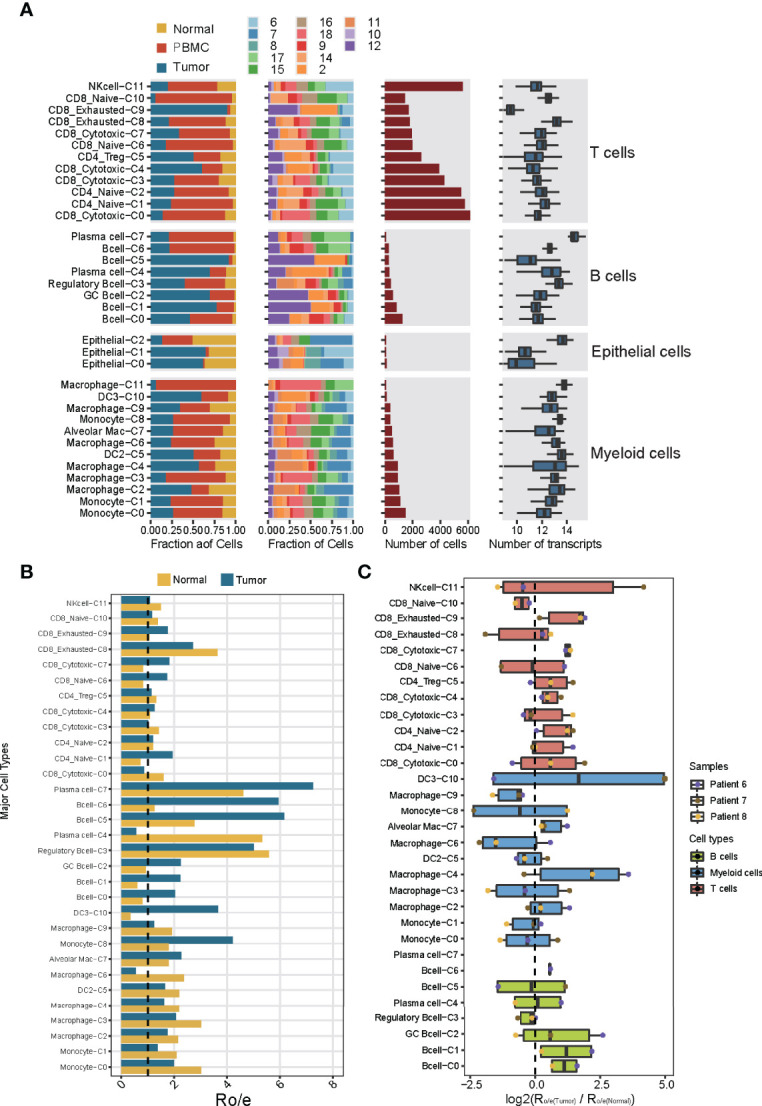
Dissection of the 32 cell types in NSCLC based on single-cell RNA sequencing. **(A)** Data on the 32 immune cell subclusters of 55,501 cells from 16 samples. Left: fractions of cells originating from each tissue and each patient. Middle: numbers of cells. Right: box plots of the numbers of genes. **(B)** Preferential localization of each cluster in tumor or normal tissue, based on the ratio of observed to expected cell numbers (RO/E). The vertical dashed line indicates a ratio of 1, with values >1 indicating enrichment in the indicated tissue. **(C)** Preferential localization of each cluster in tumor or normal tissue, based on the average ratio of observed to expected cell numbers across three patients (nos. Patient 6-8). Box plot shows log2(Ro/e(Tumor)/Ro/e(Normal)) score.

The different immune cell types differed in their distribution in the TME (**Figure 2A**), T and B lymphocytes were more concentrated in tumors than normal tissues (**Figure 2B**), which is consistent with several previous results (9, 13). Of the 32 subclusters, subcluster C9 of exhausted CD8^+^ T cells and subcluster C0 of B cells were found almost exclusively in tumors, whereas subclusters C10 of naïve CD8^+^ T cells and C3 of regulatory B cells were more abundant in normal tissues (**Figure 2C**). These differences between tumor and tissues were confirmed using multiplex immunofluorescence of formalin-fixed, paraffin-embedded sections from the same patients (**Supplementary Figure 5**). This immunostaining further showed that regulatory T cells were nearly absent from tumors but were abundant in stroma, where 90% were close to immunosuppressive PD-1^+^ cells. These observations imply suppressive immune activity within the TME.

Comparison of genes differentially expressed between the subcluster C9 of exhausted CD8^+^ cells from tumors and normal tissues showed GNLY and ITM2A were highly expressed in normal tissues, and high expression of FABP5 and RPS6 was exhibited in tumor tissues. (**Supplementary Figures 6, 7**). FABP5 gene have been reported to mediate lipid uptake and intracellular transport of CD8^+^ T cells, and was critical for the control of immune function mainly through releasing proinflammatory cytokines (14). In this way, our results may help clarify how exhausted CD8^+^ T cells influence immune activity in NSCLC.

The relative abundance of different immune cell subclusters differed substantially across tumors from different patients (**Supplementary Figure 8A**). For example, tumors from patients 2 and 12 contained abundant T and B lymphocytes, whereas the tumor from patient 7 contained few such cells and instead abundant myeloid cells. These results identify the tumor from patient 7 as an “immune-cold” tumor with striking features of T cell absence or exclusion (15). Tumors from patients 8 and 11 contained abundant B lymphocytes and myeloid cells. Tumors from patients 6 and 10 contained abundant T lymphocytes and NK cells, implying different immune cell patterns in NSCLC TME. These results also highlight the strong variation in immune cell landscape across tumors from different patients (16). This diversity may also help explain differences in prognosis: patient survival was negatively associated with the abundance of myeloid subcluster C0, and positively associated with abundances of the myeloid subcluster C4 and CD8^+^ subcluster C0 (**Supplementary Figure 8B**). Thus, analyzing the immune cell landscape in the TME may help predict response to immunotherapy.

### T/NK Cell Phenotypes Regulate Antitumor Immune Responses in NSCLC

**Figure 3:**
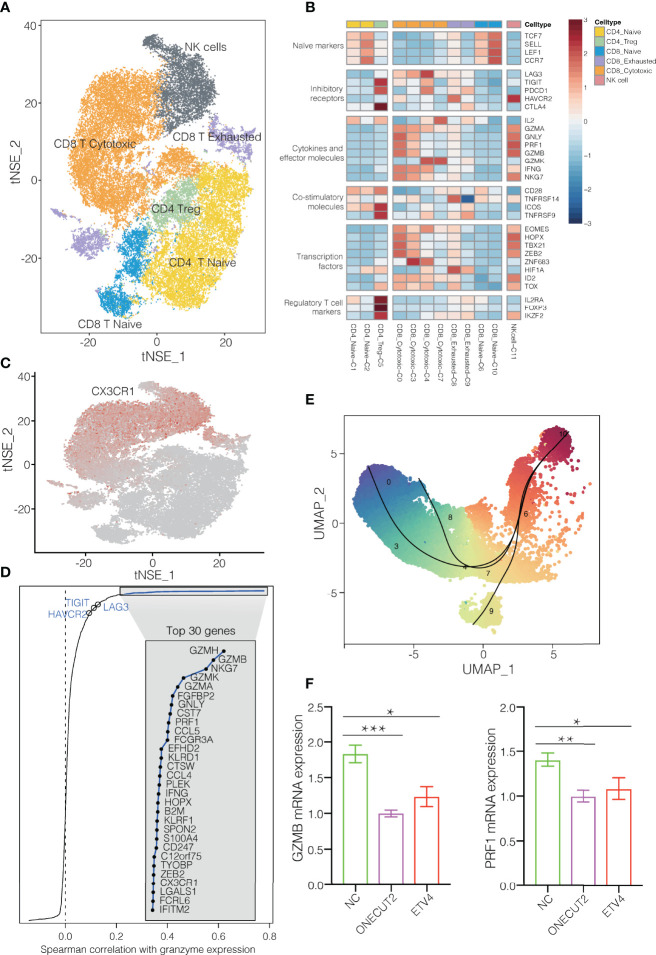
T/NK cell clusters in NSCLC. **(A)** Refined clustering was performed on 42,665 T/NK cells from tumor (n = 7), adjacent normal lung (n = 3) and peripheral blood (n = 6). These cells are color-coded by their associated clusters. **(B)** Average expression of selected T cell function-associated genes of naïve markers, inhibitory receptors, cytokines and effector molecules, co-stimulatory molecules, and Treg markers in each cell cluster. Red to blue: high to low expression. **(C)** t-SNE plot, Expression and distribution of CX3CR1 gene among cells. color-coded from gray to red according to expression. **(D)** Spearman correlation between the activity of CD8^+^ T cells, as measured by average granzyme expression (GZMA, GZMB and GZMH), and the expression of CD8^+^ T cell-specific genes. Genes encoding known immune checkpoint molecules are highlighted in blue. **(E)** Transitional relationship among 23,151 CD45^+^ CD8^+^ T cells predicted by Slingshot. Rainbow coloring from red to blue represented the begin to end of the trajectory. **(F)** Levels of mRNAs encoding ONECUT2 or ETV4 in human CD8^+^ T cells transfected with plasmids overexpressing those proteins, or with empty vector (negative control, NC). Data are mean ± SEM of three independent experiments, each conducted with triplicate samples. *P < 0.05, **P < 0.01, ***P < 0.001.

T and NK cells are the most abundant immune cells in the NSCLC TME. We classify the 42,665 T/NK cells into subclusters C1 and C2, which contained naïve CD4^+^ T cells; subcluster C5, CD4^+^ regulatory T cells; subclusters C6 and C10, naïve CD8^+^ T cells; subclusters C0, C3, C4 and C7, cytotoxic CD8^+^ cells; subclusters C8 and C9, exhausted CD8^+^ T cells; and subcluster C11, NK cells (**Figures 1D**, **3A**). Comparison of gene expression among these subclusters (**Figure 3B**) showed that subclusters C0 and C3 of cytotoxic CD8^+^ T cells highly expressed cytokines and their effector molecules, including the chemokine receptor CX3CR1 (17) and the cytotoxicity-associated genes PRF1, GZMA and GZMB (18) (**Figure 3C** and **Supplementary Figure 9**). Thus, these two subclusters may be the major populations of cytotoxic CD8^+^ T cells in NSCLC.

In contrast, the subclusters C6 and C10 of naïve CD8^+^ cells strongly expressed the well-defined naïve markers CCR7, TCF7, LEF1 and SELL. The subcluster C8 of exhausted CD8^+^ T cells expressed high levels of the inhibitory markers PDCD1, LAG3 and HAVCR2, but low levels of GZMA and GZMK. During the progression towards late dysfunctionality, classic CD8^+^ T cell effector function- associated genes, such as PRF1 and GZMB remains high (19, 20). Subclusters C0, C3 and C4 of cytotoxic CD8^+^ T cells expressed immune checkpoint molecules, particularly express LAG3 and PDCD1 (**Figure 3B**). LAG3 was mainly expressed in CD8^+^ exhausted T cells, which is consistent with previous findings (20). In fact, expression of LAG3 and HAVCR2 varied positive with expression of cytotoxic granzymes GZMA and GZMH (**Figure 3D**). These results suggest an activation-dependent exhaustion phenotype mediated by LAG3 in NSCLC. Thus, these two checkpoint molecules may be potentially therapeutic targets. The subcluster C5 of regulatory T cells strongly expressed the exhaustion markers CTLA4, HAVCR2 and TIGIT (**Figure 3B**), in contrast to subclusters C1 and C2 of naïve CD4^+^ T cells. Furthermore, these T cell clusters appeared to exhibit distinct tissue distributions. Subcluster C0 of cytotoxic CD8^+^ cells localized predominantly in blood, whereas subcluster C9 of exhausted CD8^+^ T cells were more abundant in tumors. Subcluster C9 may be tumor-specific subtypes, which implies an exhaustion expression program in NSCLC (**Supplementary Figure 10**). Altogether, the changes in cellular composition and gene expression phenotype of T cells confirmed the direction of tumor immunity towards immune suppression (21, 22).

Pseudotime analysis based on Slingshot (23) suggested that CD8^+^ T cells followed a differentiation trajectory extending from subcluster C10 of naïve CD8^+^ cells to subcluster C0 of cytotoxic CD8^+^ cells as well as subclusters C8 and C9 of exhausted CD8^+^ T cells (**Figure 3E**). Consistent with their exhausted phenotype, the CD8^+^ T cells in subcluster C9 showed lower expression of cytotoxicity-associated genes GZMH, GZMA, and PRF1 than subcluster C0 of cytotoxic CD8^+^ T cells (**Supplementary Figure 9**). In addition, gene set variation analysis showed that the exhausted cells in subcluster C9 expressed genes involved in tryptophan metabolism (**Supplementary Figure 11**), which impair antitumor immune responses (19), whereas the cytotoxic cells in subcluster C0 cells expressed genes involved in antigen processing, antigen presentation, and T cell receptor signaling.

Pseudotime analysis suggested that conventional CD4^+^ T cells followed a developmental trajectory extending from subcluster C2 of naïve CD4^+^ T cells to subclusters C1 of naïve CD4^+^ T cells and C5 of exhausted CD4 regulatory T cells (**Figure 3B** and **Supplementary Figure 12**). Subcluster C5 strongly expressed the exhaustion markers PDCD1, CTLA4 and TIGIT (**Supplementary Figure 13**), as well as the T follicular helper markers CXCL13 and ICOS (**Figure 3B** **)**, consistent with a previous finding that exhausted CD4^+^ T cells tend to adopt a T follicular helper phenotype (24).

To identify transcription factors that might regulate these various CD8^+^ T cell subclusters, we used single-cell regulatory network inference and clustering (11), which identified the transcription factor ONECUT2 to be highly expressed in subcluster C8 of exhausted CD8^+^ T cells and the transcription factor ETV4 to be strongly expressed in subcluster C9 of exhausted CD8^+^ T cells (**Supplementary Figure 14**). Consistent with this analysis, we found that overexpressing either transcription factor in CD8^+^ T cells from the blood of NSCLC patients downregulated GZMB and PRF1 expression (**Figure 3F**). These findings identify ONECUT2 and ETV4 as potential regulators of CD8^+^ T cell exhaustion in NSCLC. Conversely, the transcription factors BACH1 and RUNX3 were upregulated in subcluster C0 of CD8^+^ cytotoxic T cells (**Supplementary Figure 14**). Thus, these factors may help drive cytotoxic immune responses in NSCLC (25–27).

### Diverse B Cell Subtypes in NSCLC

**Figure 4:**
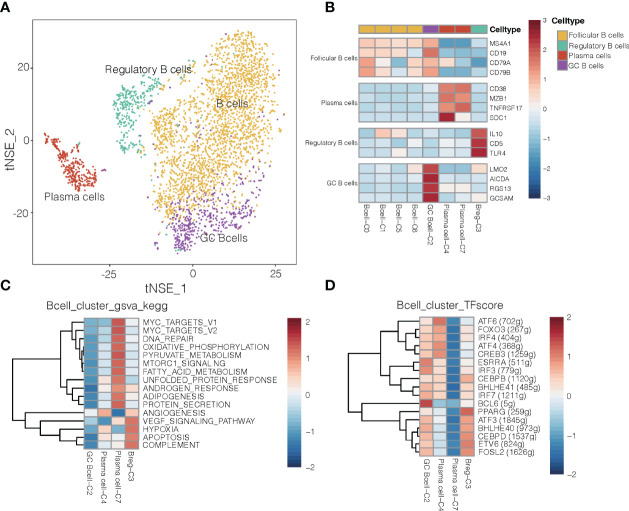
B cell clusters in NSCLC. **(A)** Refined clustering was performed on 3,958 B cells from tumor (n = 7), adjacent normal lung (n = 3) and peripheral blood (n = 6). These cells are color-coded by their associated clusters and cell types. GC, germinal center. **(B)** Average expression of selected B cell type marker genes after normalization by z-score within each cell cluster. **(C)** Heatmap showing differences in pathway activity scores among regulatory B cells, plasma cells and germinal center B cell clusters of B cells, based on gene set variation analysis. Activity scores were normalized. **(D)** Heatmap of gene expression regulation by transcription factors using SCENIC for the B cells. TF activity is scored using AUCell, and the scores are normalized.

Given that B cells infiltrate lung cancer at all stages of the disease and contribute to anti-tumor immunity (28), we detected 3,958 B cells and divided them into eight clusters (**Figures 4A, B**). One cluster contained germinal center B cells (GC B cells) that strongly expressed LMO2, AICDA and RGS13, whereas another contained regulatory B cells that expressed IL-10, CD5 and TRL4 and that were more abundant in tumors (**Supplementary Figure 15**) than in normal tissues and in blood (29). Polarization of B cells into a phenotype that secreted abundant IL-10 was associated with strong expression of inflammatory signals. One cluster was characterized as plasma cells, which strongly expressed CD38, SDC1, MZB1 and TNFRSF17 (**Figure 4C**). It has been reported that GC B cells with relatively high affinity could be directed to become plasma cells (30).

Differences in pathway activities and TFs among the different B cell subtypes are shown in **Figures 4C, D**, respectively. Cluster C3 of regulatory B cells and cluster C4 of plasma cells showed activation of angiogenesis pathways, while cluster C2 of GC B cells showed upregulation of the transcription factor BCL6 (**Figure 4D**), which enhances B cell responses to external stimuli. Cluster C4 of plasma cells showed upregulation of the transcription factor ATF4 and ATF6, which helps drive immunoglobulin production (31). These transcription factors may help drive the differences in gene expression observed between GC B cells and plasma cells in NSCLC.

In mouse models of cancer, tumor-associated B cells promote antitumor inflammation (32), but they can also dampen responses to antitumor therapies that depend on T cells (33). Cluster C3 of regulatory B cells, which were more abundant in tumors than in normal tissues or blood (**Supplementary Figure 15**), showed upregulation of pathways related to angiogenesis, VEGF signaling and hypoxia (**Figure 4C**). Thus, this cluster may help promote NSCLC progression. Regulatory B cells in our samples expressed some different genes depending on their location: while B cells in tumors strongly expressed HLA genes, which are related to antigen presentation, B cells in normal tissues strongly expressed genes related to energy metabolism (**Supplementary Figure 16**). Such spatial differences may provide insights into how regulatory B cells influence the TME and thereby cancer progression.

### Myeloid Cell Subtypes Show Tissue-Specific Patterns in NSCLC

**Figure 5:**
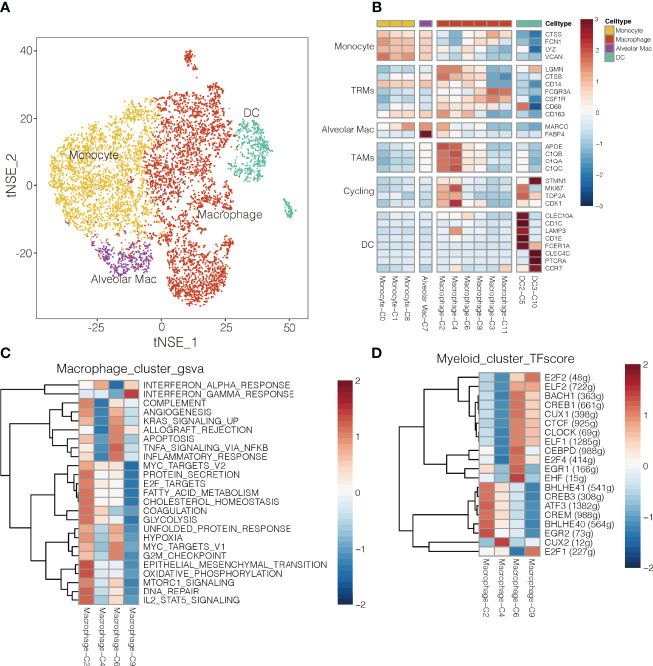
Myeloid cell clusters in NSCLC. **(A)** Refined clustering was performed on 8,026 myeloid cells from tumor (n = 7), adjacent normal lung (n = 3) and peripheral blood (n = 6). These cells are color-coded by their associated clusters and cell types DC, dendritic cell; Mac, macrophage **(B)** Average expression of selected myeloid cell marker genes and function-associated genes, after normalization by z-score within each cell cluster. RTMs, resident tissue macrophages; TAMs, tumor-associated macrophages; DCs, dendritic cells; Alveolar Mac, alveolar macrophages. **(C)** Heatmap showing differences in pathway activity scores among clusters of macrophages, based on gene set variation analysis. The scores of pathways are normalized. **(D)** Heatmap of gene expression regulation by transcription factors using SCENIC for the macrophages. TF activity is scored using AUCell, and the scores are normalized.

To begin to clarify the heterogeneity of myeloid cells in the TME and their potential roles in cancer, we characterized gene signatures of 12 subsets (**Figures 1D**, **5A**). The 8,026 myeloid cells were clustered into monocytes, macrophages, and DCs. Monocytes expressed a unique set of genes, which included CTSS, FCN1, LYZ and VCAN (**Figure 5B**), and they were more abundant in blood than in tumors or normal tissues (**Supplementary Figure 17**). These results are consistent with the known roles of monocytes in regulating inflammation and responding to bacterial infection in the blood, from which they can infiltrate tissues and differentiate into tissue-resident CD14^+^ CD16^-^ macrophages (34). Two DC subsets strongly expressed DC markers, and they were more abundant in tumor and normal tissue than in blood (**Figure 5B** and **Supplementary Figure 17**). The DC2 subtype preferentially expressed classic markers of conventional type 2 DCs, including CD1E, FCER1A, LAMP3 and CLEC10A. LAMP3 pression has also been linked to activation and migration of DCs (3), and this protein was also highly expressed by cluster C4 of macrophages.

The macrophage clusters were identified based on their high expression of CSF1R (CD115), FCGR3A (CD16), and CD86. Clusters C6 and C9 were more abundant in normal tissue, whereas clusters C2 and C4 were more abundant in tumors (**Supplementary Figure 17**); therefore, clusters C6 and C9 were designated as TAMs (35), which strongly expressed APOE, C1QA and C1QB (36), as well as the type I transmembrane glycoproteins, MKI67 and CDK1 (**Figure 5B**). Clusters C3 cells were more abundant in blood, so they were designated as wandering macrophages (8). Cluster C2 of TAMs showed upregulation of pathways involved in inflammatory responses, TNF-α-induced proliferation and production of reactive oxygen species (**Figure 5C**). These pathways are hallmarks of the M2-like, pro-tumoral subtype of macrophages in mouse models of cancer (37). Indeed, cluster C2 in our study showed strong enrichment of angiogenesis, glycolysis, and hypoxia pathways, based on gene set variation analysis (**Figure 5C** and **Supplementary Figure 18**). These results suggest a strong tumor-promoting phenotype of cluster C2 TAMs (38). Consistently, cluster C2 strongly expressed S100A8 and S100A9 (**Supplementary Figure 19**), which may promote cell proliferation while inhibiting cell differentiation and apoptosis (39). In contrast, cluster C4 of TAMs showed upregulation of IFN-α response pathways.

Single-cell regulatory network inference and clustering identified several transcription factors that may help drive the observed differences in gene expression among the myeloid cell types in NSCLC (**Figure 5D**). Cluster C2 of macrophages showed upregulation of BHLHE40, which helps drive breast cancer metastasis (27), and those expression levels correlated inversely with survival (**Supplementary Figure 20**). Cluster C9 of macrophages showed upregulation of E2F1 (**Figure 5D**), inducing their repolarization into proinflammatory M2-like macrophages (40), and thereby weaken T cell proliferation and antitumor activity (41).

## Discussion

One of the major obstacle to cancer immunotherapy is the heterogeneous composition of immune cells within the TME. Using single-cell transcriptomics on tissues and bloods from NSCLC patients, we constructed an immune atlas of the disease that highlights how much gene expression and phenotype depend on whether the immune cell is located within the tumor, in normal tissue, or in the blood.

Tumors are thought to evade natural immune surveillance either by immune escape or by active suppression of immune responses. We identified several T cell subtypes expressing cytokines and chemokines, indicative of ongoing immune responses. At the same time, some of those subtypes were exhausted or regulatory T cells showing immunosuppressive phenotypes. From the differential gene expression analysis, we showed an activation-dependent exhaustion phenotype mediated by LAG3 in NSCLC. This phenomenon has been demonstrated by previous studies in NSCLC that during the progression towards late dysfunctionality, classic CD8^+^ T cell effector function- associated genes remains high (19, 20). In a subsequent study, the transcription factor ONECUT2 and ETV4 was found to be overexpressed and to downregulated GZMB and PRF1 expression, thus leading to dysfunctional T cells. Therefore, the ONECUT2 and ETV4 transcription factor may be useful target for checkpoint inhibition.

Of note, overall survival in NSCLC appears to depend on effective infiltration of tumors by B cells (42), and stimulating B cells with appropriate ligands can inhibit tumor growth and lung metastasis in mouse models. Thus, immunotherapies based on B cells may be effective. Our results suggest a potential approach to B cell therapy: exploit the tumor-promoting features of regulatory B cells to inhibit downstream immunosuppressive pathways. On the other hand, our results are consistent with the notion that lymphocytes possess relatively stable phenotypes, whereas myeloid cells can be more “plastic” depending on their location in the TME (21). We were able to identify 12 subtypes myeloid cells from advanced NSCLC besides T/NK and B cells, the majority of which are consistent with previous studies (20). We focused on the tumor associated macrophages, which were highly plastic and display a variety of phenotypes (16). Consistently, we observed greater phenotypic complexity among TAMs, including one TAM population in which inflammatory responses, TNF-α-induced proliferation and production of reactive oxygen species were upregulated. Additionally, high expression of the BHLHE40 of macrophages significantly predicted a poor prognosis in NSCLC.

Finally, characterization of the TME on single-cell resolution can provide insight on possible novel therapeutic targets. It remains an open question to which extent tumor cells shape their microenvironment and to which extent the microenvironment affects tumor cells. More efforts need to be complemented by translational studies to identify critical mechanisms in this complex network that determine tumor response to targeted or immune therapies in the clinical context. In the future, other single-cell approaches comprising spatial information, and surface protein expression will help to complete the picture.

## Conclusion

In summary, our work represents a new resource providing a comprehensive single-cell transcriptome atlas of the multicellular ecosystem of NSCLC TME. Our high-resolution immune landscape of NSCLC has identified ONECUT2 and ETV4 transcription factors as potential drivers of CD8^+^ T cell exhaustion. It also reveals exhausted subtypes of TAMs and regulatory B cells that may immunosuppress the TME. These insights from the present work may help identify novel therapeutic targets and biomarkers of therapy response in NSCLC. Some of our findings are unreported and will need further functional validation. Despite this limitation, it can serve as valuable resources and a proof-of-concept study for future research to identify biomarkers and targets for treatment and enable personally tailored therapeutic decisions for patients with advanced NSCLC.

## Data Availability Statement

The generated scRNA-seq data in this study have been deposited to Genome Sequence Archive (GSA) in BIG Data Center, Beijing Institute of Genomics (BIG) under accession number HRA002473. The transcriptome data of TCGA LUAD were collected from the following web-links https://portal.gdc.cancer.gov/projects/TCGA-LUAD. The human-specific databases for RcisTarget were downloaded from (https://resources.aertslab.org/cistarget/databases/homo_sapiens /hg19/refseq_r45/mc9nr/gene_based/hg19-500bp-upstream-7species.mc9nr.feather) and (https://resources.aertslab.org/cistarget/databases/homo_sapiens/hg19/refseq_r45/mc9nr/gene_based/hg19-tsscentered-10kb-7species.mc9nr.feather).

## Ethics Statement

The studies involving human participants were reviewed and approved by Institutional Review Board of West China Hospital of Sichuan University. The patients/participants provided their written informed consent to participate in this study. Written informed consent was obtained from the individual(s) for the publication of any potentially identifiable images or data included in this article. This study protocol was approved by the Institutional Review Board of West China Hospital of Sichuan University (Ethics: Project identification code: 2018.270).

## Author Contributions

ZW performed single-cell RNA sequencing experiments. YZ and YL performed sequencing and processed the data. QZ, KT, CY, KL, CC, and YMC performed bioinformatic analyses. YMC, WXL, XX, and JT obtained patient consent and collected the samples. XX and YL assisted in participant selection, consent, clinical information and procurement of tissue. YN and GZ performed fresh tissue dissociations, immunostaining, microscopy and imaging. ZW, WZ, GC, DX, and WML provided clinical insights. ZW and QZ analyzed and interpreted the data. ZW and QZ conceived the study and wrote the manuscript. All authors contributed to the article and approved the submitted version.

## Funding

This work was supported by grants from the National Natural Science Foundation of China to W. Li (81871890, 91859203) and to Z. Wang (81802300, 32170592), grants from the Sichuan Science and Technology Program to L. Li (2019YFS0339) and M. Yang (2020YFS0573), and a grant from the Chengdu Science and Technology Program to W. Zhang (2017CY0200017GX).

## Conflict of Interest

The authors declare that the research was conducted in the absence of any commercial or financial relationships that could be construed as a potential conflict of interest.

## Publisher’s Note

All claims expressed in this article are solely those of the authors and do not necessarily represent those of their affiliated organizations, or those of the publisher, the editors and the reviewers. Any product that may be evaluated in this article, or claim that may be made by its manufacturer, is not guaranteed or endorsed by the publisher.
