# Supplementary material for: Single-Cell Transcriptomics of Immune Cells Reveal Diversity and Exhaustion Signatures in Non-Small-Cell Lung Cancer
Source: Front Immunol. 2022 Jul 6;13:854724. doi: 10.3389/fimmu.2022.854724 (PMC9299430; doi:10.3389/fimmu.2022.854724)
Supplement: Supplementary file 1 [file DataSheet_1.docx]

Supplementary Material


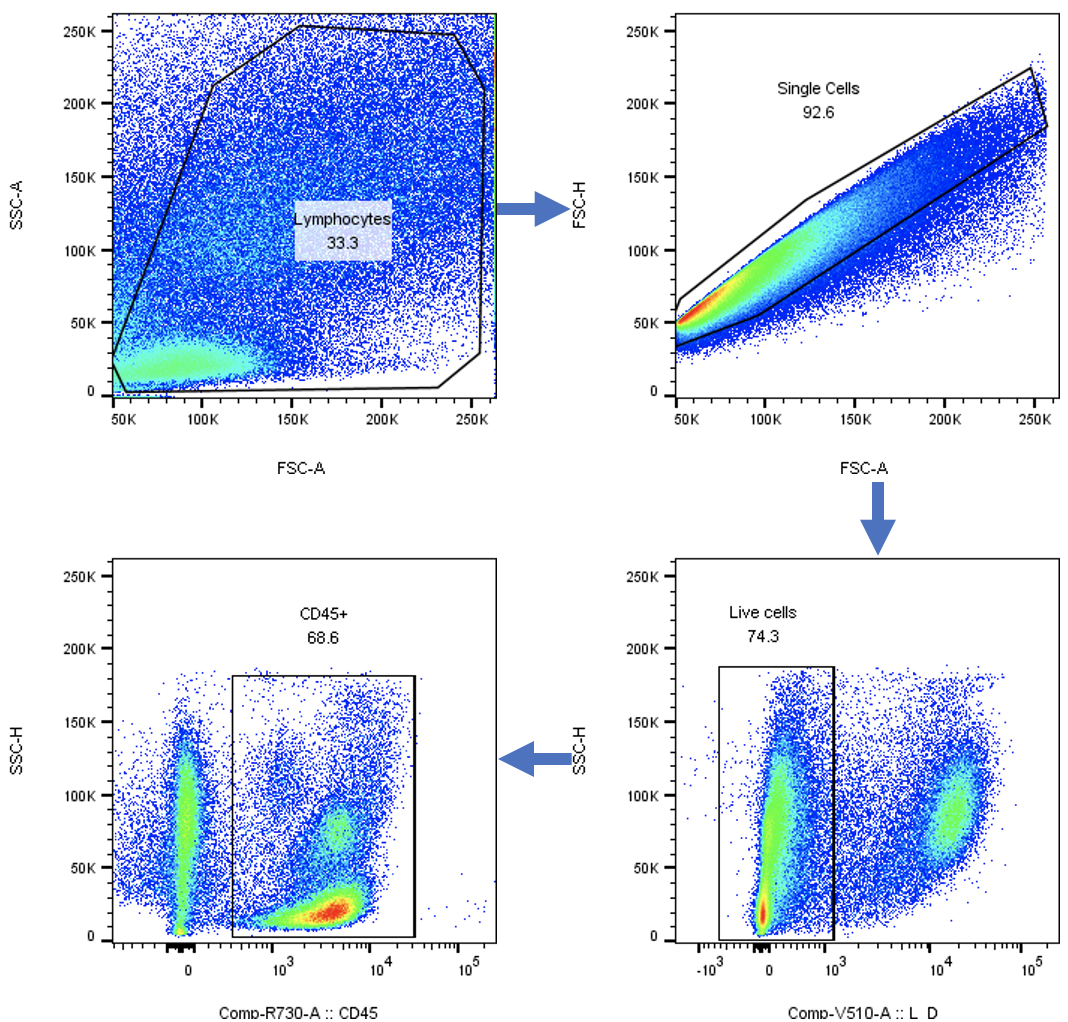


**Supplementary Figure 1**. Representative flow cytometry plots of gated live CD45^+^ immune cells for construction of single-cell RNA sequence libraries. Within each plot, the description and proportion of the boxed cell population are indicated.


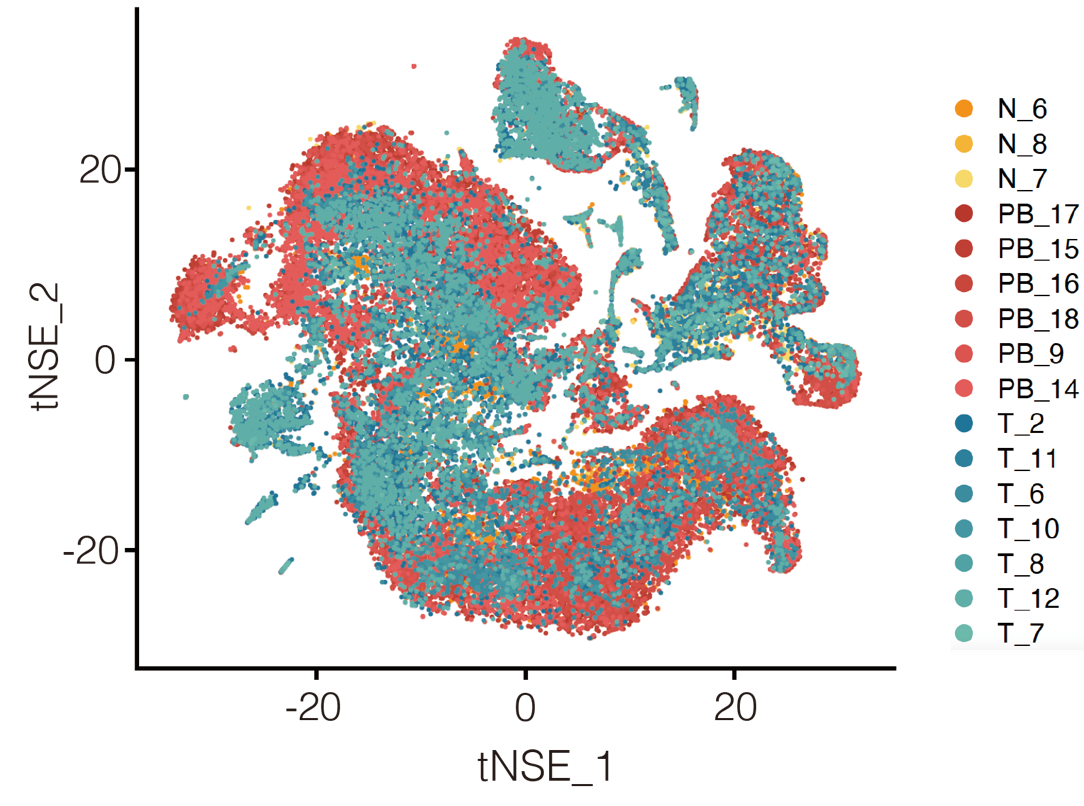


**Supplementary Figure 2.**  t-SNE plot of 55,501 CD45^+^ immune cells color-coded based on the type of tissue and patient number. (Yellow to orange: N, adjacent normal tissue; Red: PB, peripheral blood; Blue: T, tumor).


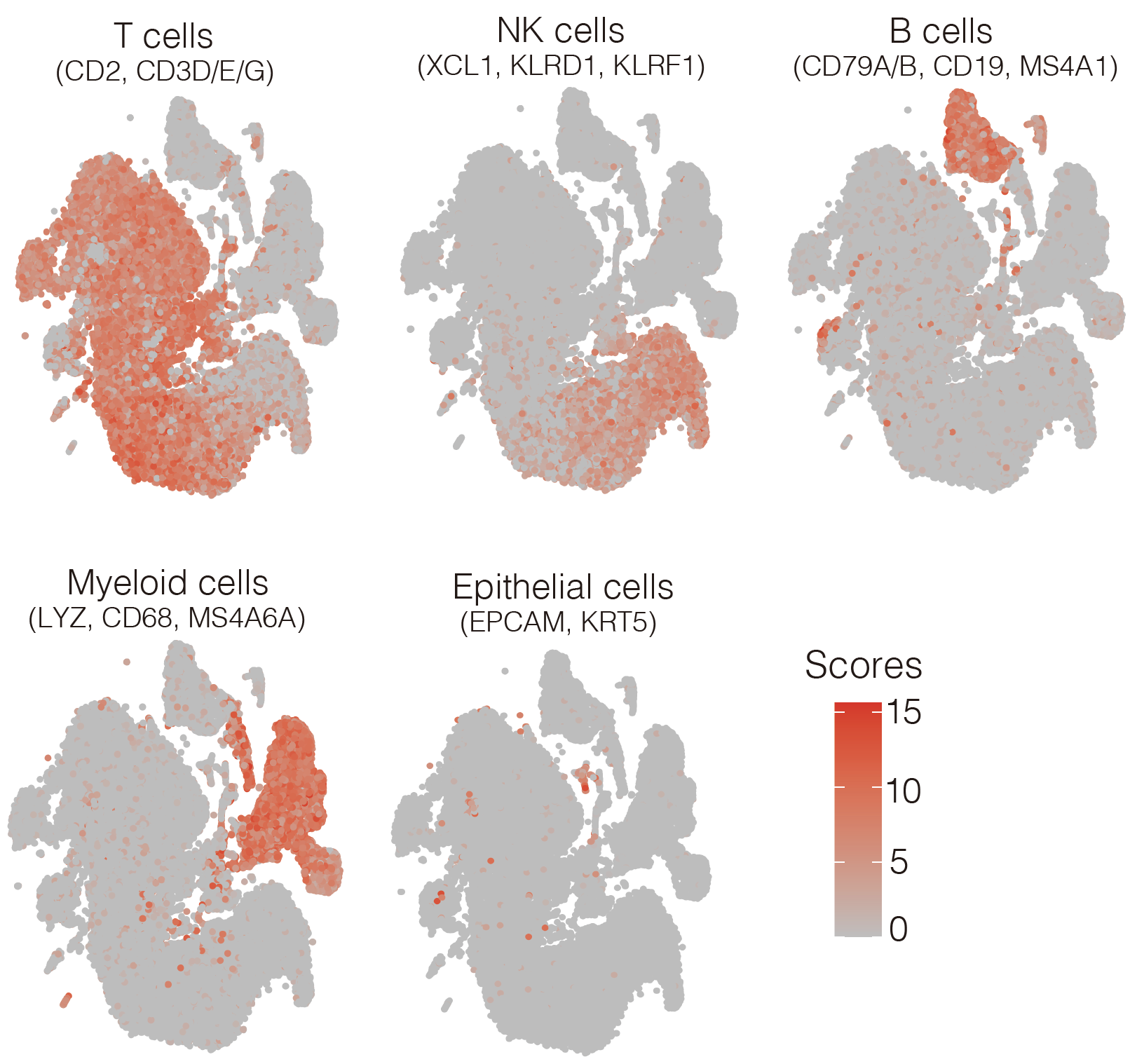


**Supplementary Figure 3.** t-SNE plots, color-coded from gray to red to reflect expression of marker genes for the indicated cell type. (Epithelial cell markers, EPCAM, KRT19, KRT18, KRT5, and KRT15; Myeloid cell markers, LYZ, CD68, MS4A6A, CD1E, IL3RA, and LAMP3; T cell markers, CD2 and CD3D/E/G; NK cell markers, XCL1, KLRD1 and KLRF1; B cell markers, CD79A/B, CD19, and MS4A1).


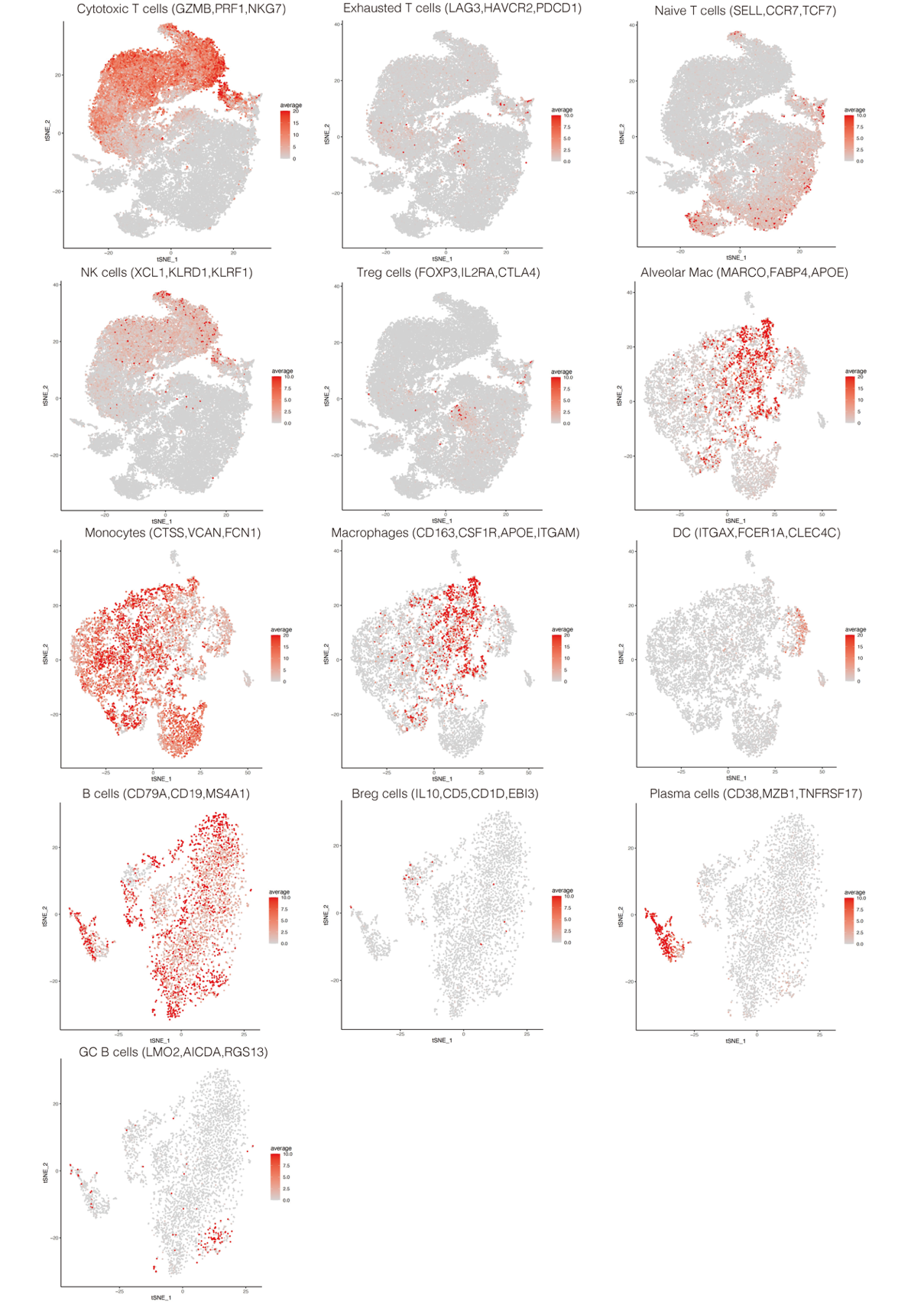


**Supplementary Figure 4.** t-SNE plots, color-coded from gray to red to reflect expression of marker genes for the indicated cell types. Marker genes for each subset were listed in the picture above.


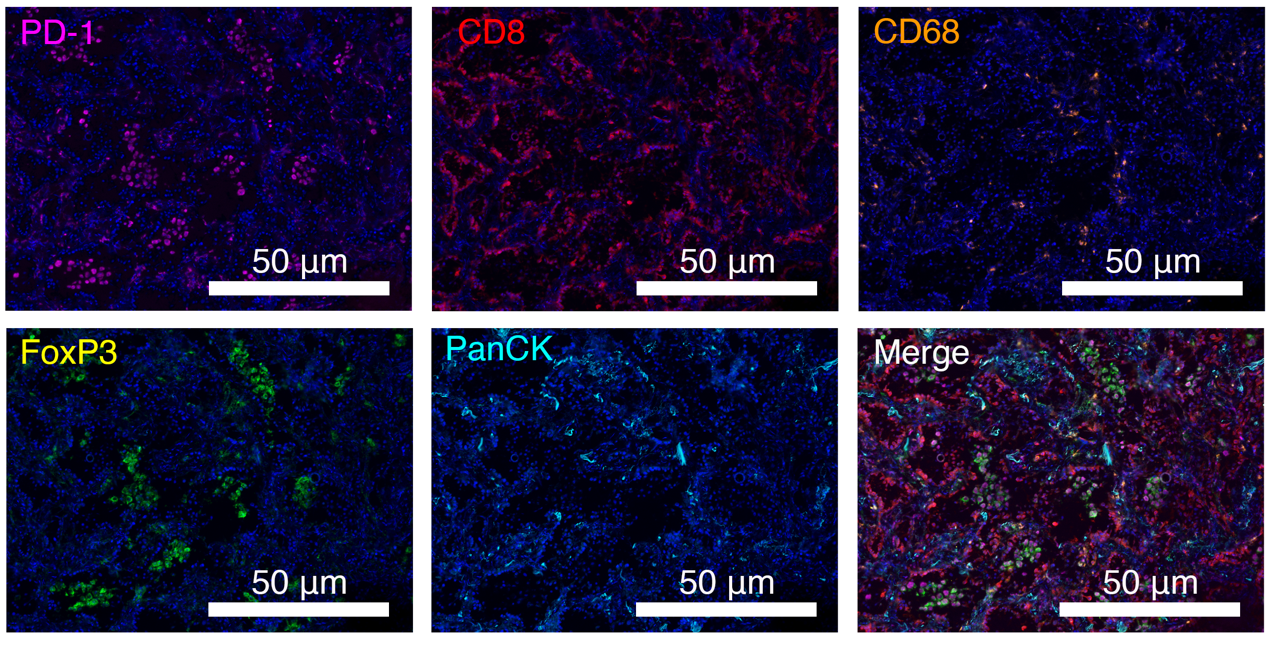


**Supplementary Figure 5**. Representative multiplexed staining against cytokeratin (cyan), CD68^+^ macrophages (orange), FoxP3 regulatory T cells (yellow), CD8^+^ T cells (red), PD-1 cells (magenta), or PD-L1 cells (green) on tumor tissues of NSCLC. Each staining in panels come from three samples. Nuclei were stained blue with DAPI. Scale bar, 50 μm.

­
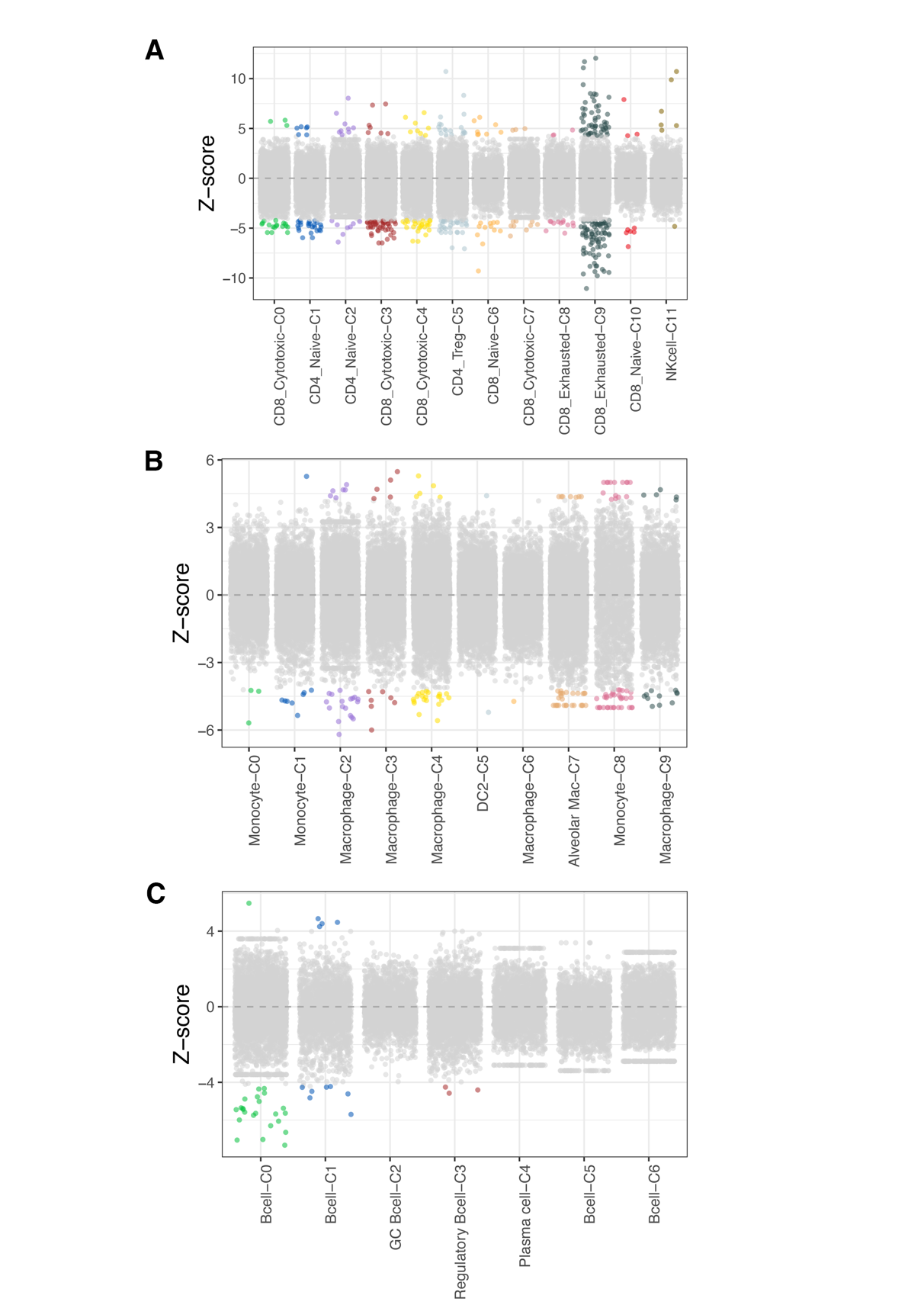


**Supplementary Figure 6.** Tissue-dependent differences in gene expression for each cell type. Each dot represents the differential expression z-score of a gene. (a) T/NK cells. (b) Myeloid cells. (c) B cells. Dots with Bonferroni-corrected P < 0.05 are in color.


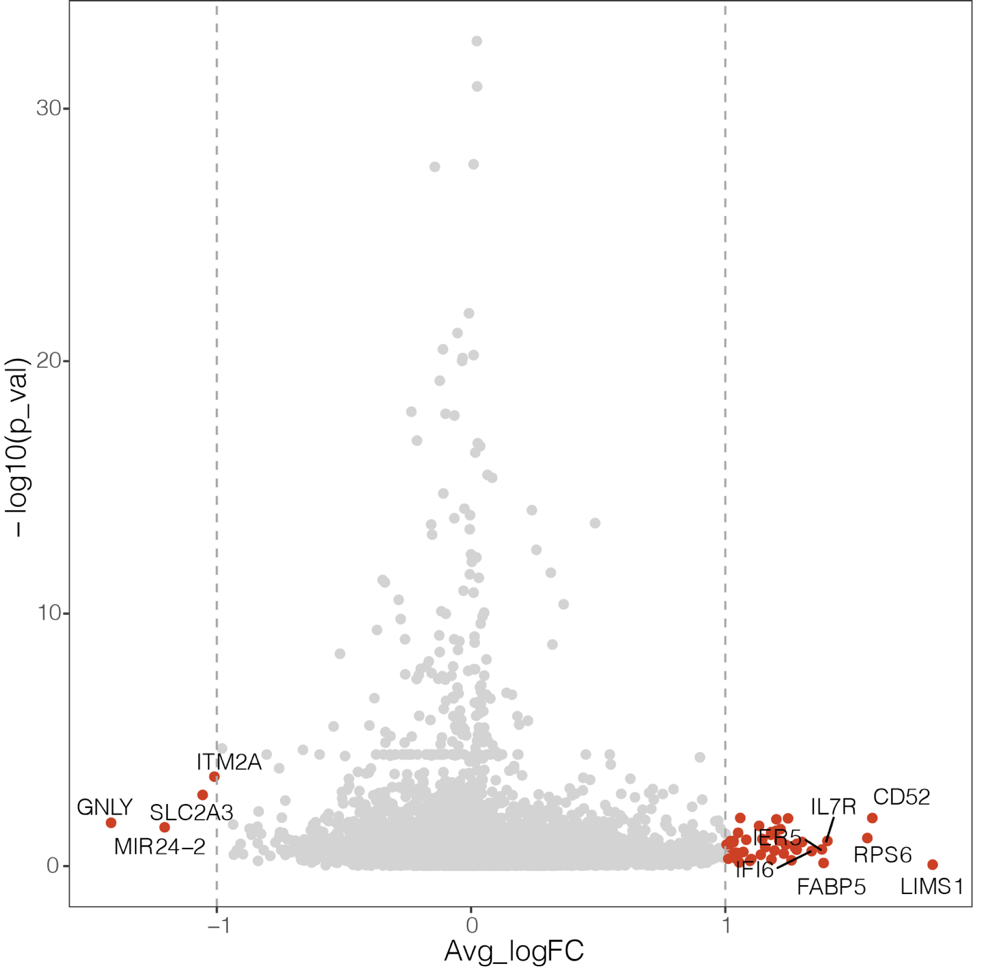


**Supplementary Figure 7.** Volcano plot showing genes in cluster C9 of exhausted CD8^+^ T cells that were differentially expressed between lung tumors and adjacent normal lung tissues. Difference between percentage of cells expressed in two subset was plotted against log fold change of p-value. FC, fold change.


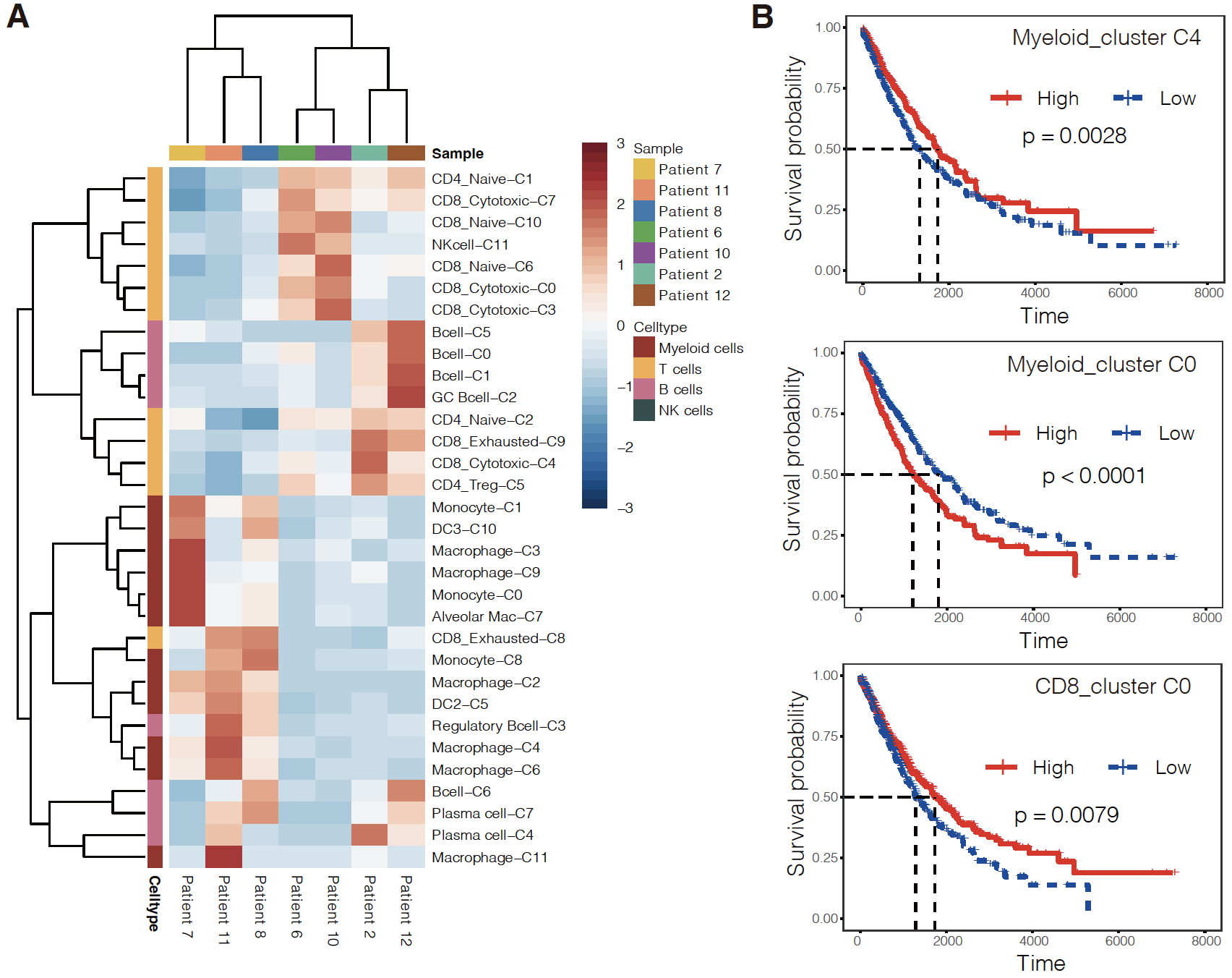


**Supplementary Figure 8.** Immune cell clusters in NSCLC patients, and correlations between immune signatures and survival. **(A)**. Heatmap showing tumor samples in each cell subtype. **(B)**. Kaplan–Meier curves showing survival for patients showing low or high expression of genes specific to cluster 0 of cytotoxic CD8^+^ or cluster 0 of monocytes and cluster 4 of Macrophage.


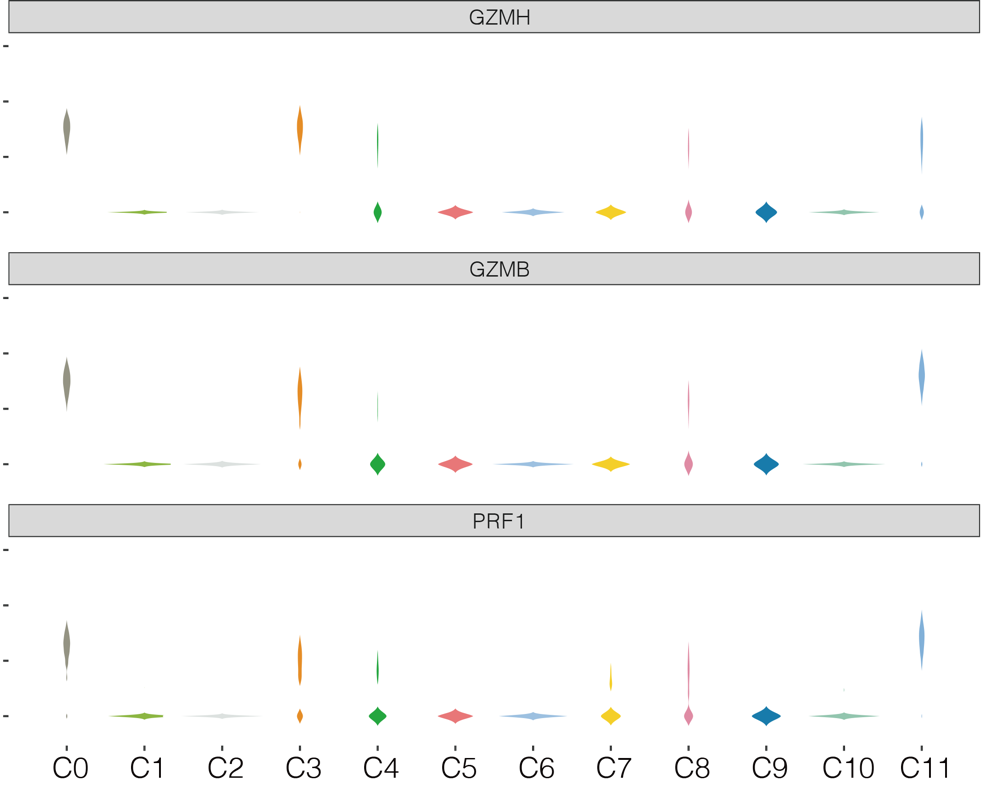


**Supplementary Figure 9.** Violin plots showing average expression of the genes associated with cytotoxicity (GZMH, GZMB and PRF1) in the T/NK cell clusters.


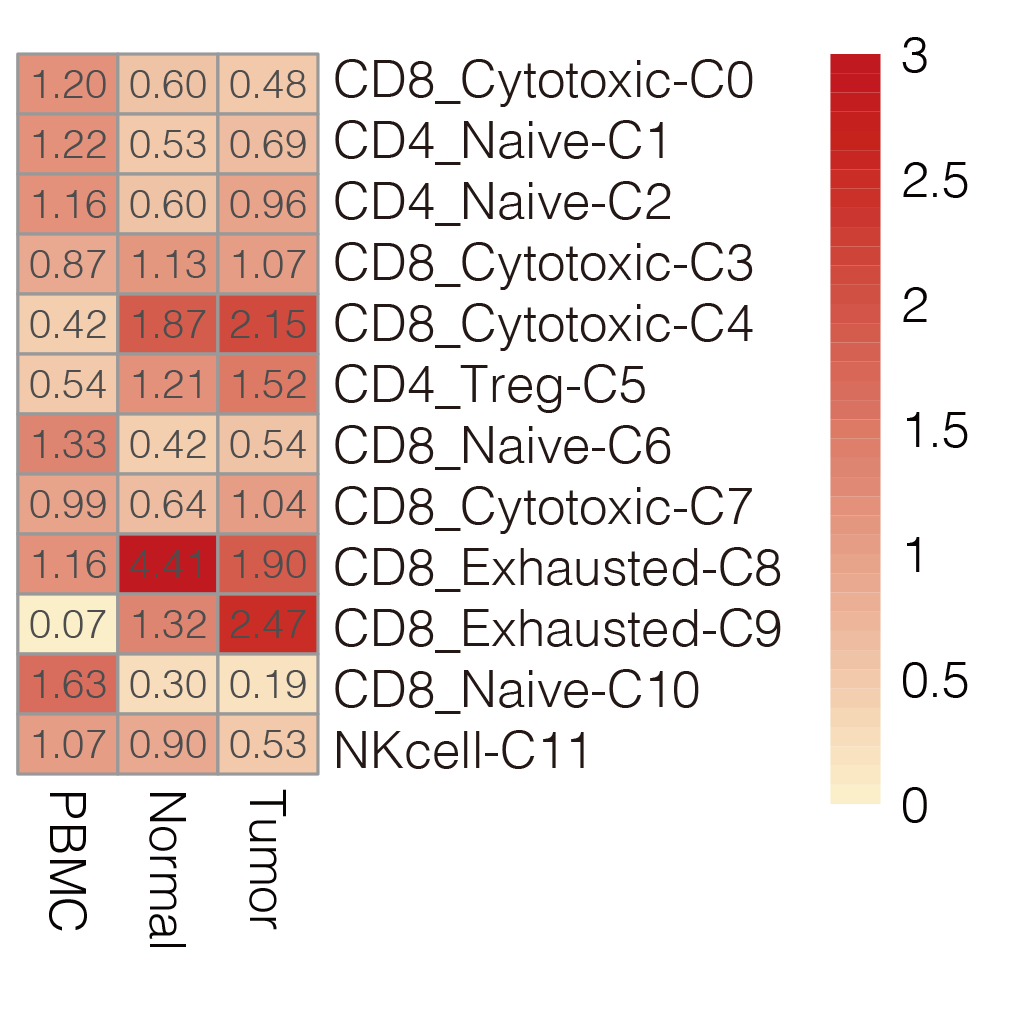


**Supplementary Figure 10**. Tissue distribution pattern of clusters of T/NK cells, based on the ratio of cell numbers (Ro/e) in each tissue. PBMC, peripheral blood mononuclear cell.


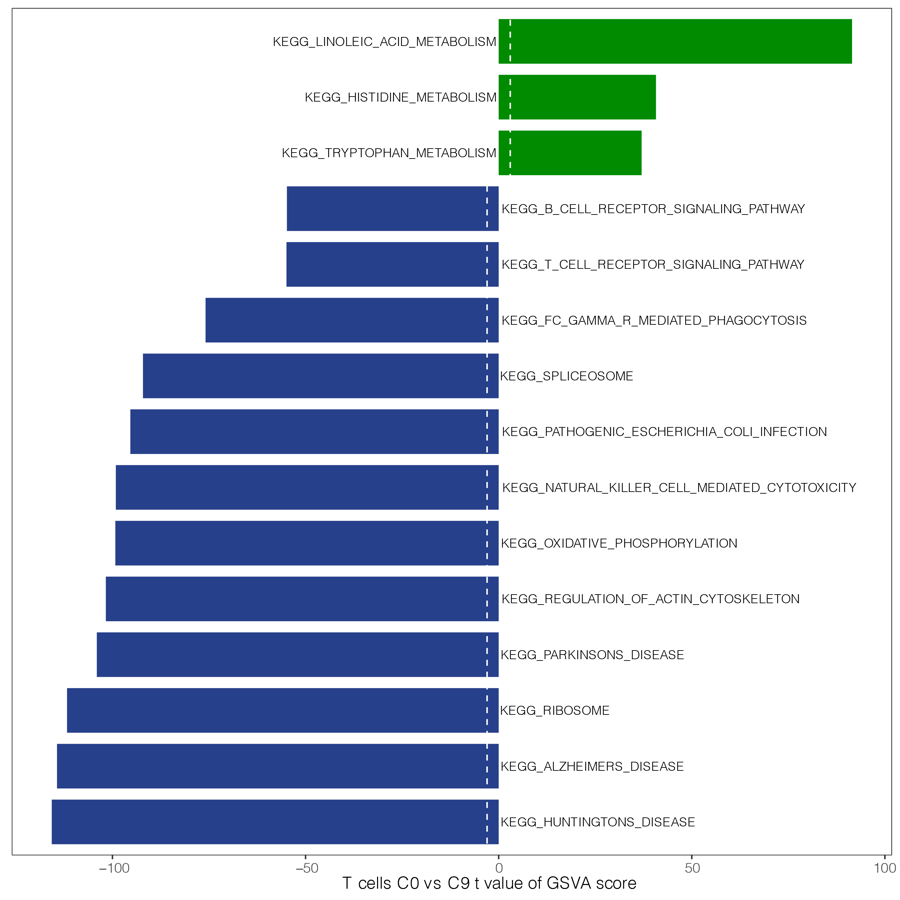


**Supplementary Figure 11.** Differences in pathway activity scores per cell between cluster C0 of cytotoxic CD8^+^ cells (n = 6154) and cluster C9 of exhausted CD8^+^ cells (n = 1689 cells), based on gene set variation analysis. Shown are t values from a linear model, corrected for patient of origin. Green: cluster C9 of exhausted CD8+ cells; Blue: cluster C0 of cytotoxic CD8^+^ T cells.


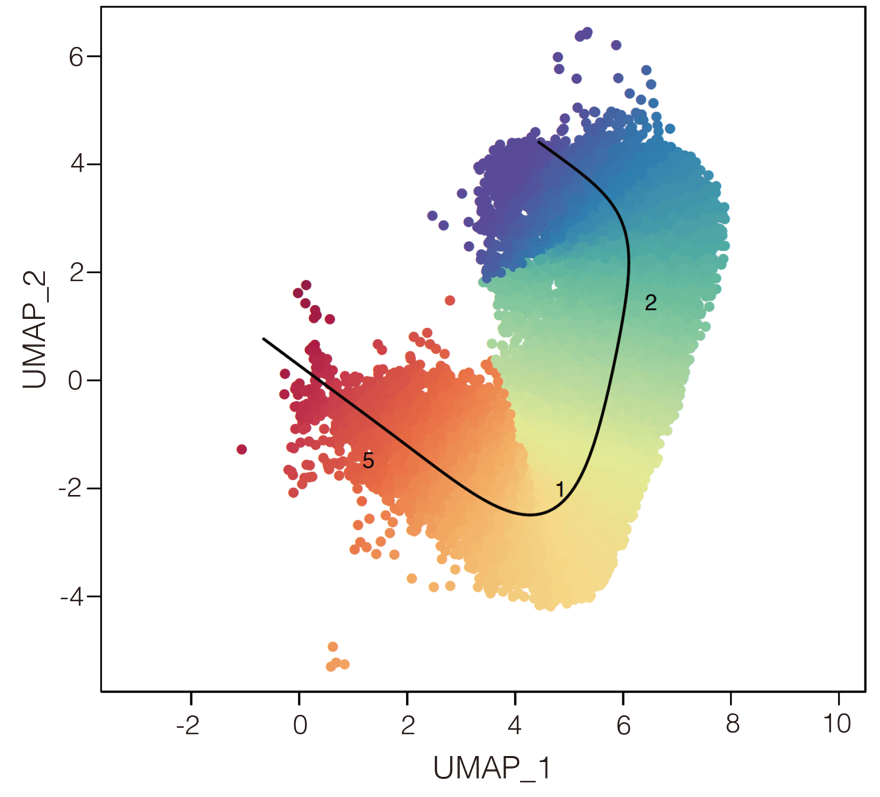


**Supplementary Figure 12**. Transitional relationship among 13,900 CD45^+^ CD4^+^ T cells predicted by Slingshot. Rainbow coloring from red to blue represented the begin to end of the trajectory. (1 represents cluster 1; 2 represents cluster 2; 5 represents cluster 5)

**Supplementary Figure 13.** Violin plots showing average expression of the genes associated with exhaustion markers (CTLA4, HAVCR2 and TIGIT) in CD4^+^ T cell clusters.


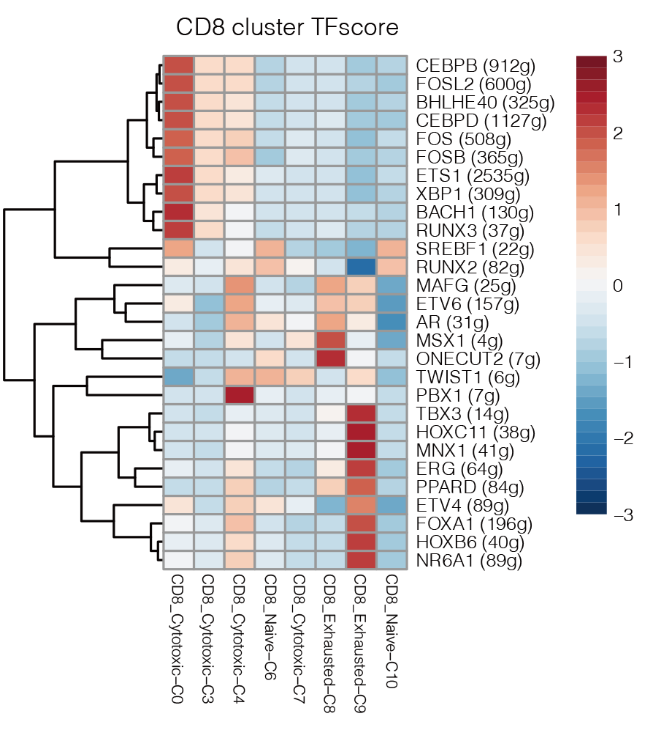


**Supplementary Figure 14.** Heatmap showing the activity of transcription factors (TFs) in each CD8^+^ T subtype, as estimated using SCENIC.





**Supplementary Figure 15**. Tissue distribution pattern of clusters of B cells, based on the ratio of cell numbers (Ro/e) in each tissue. PBMC, peripheral blood mononuclear cell.


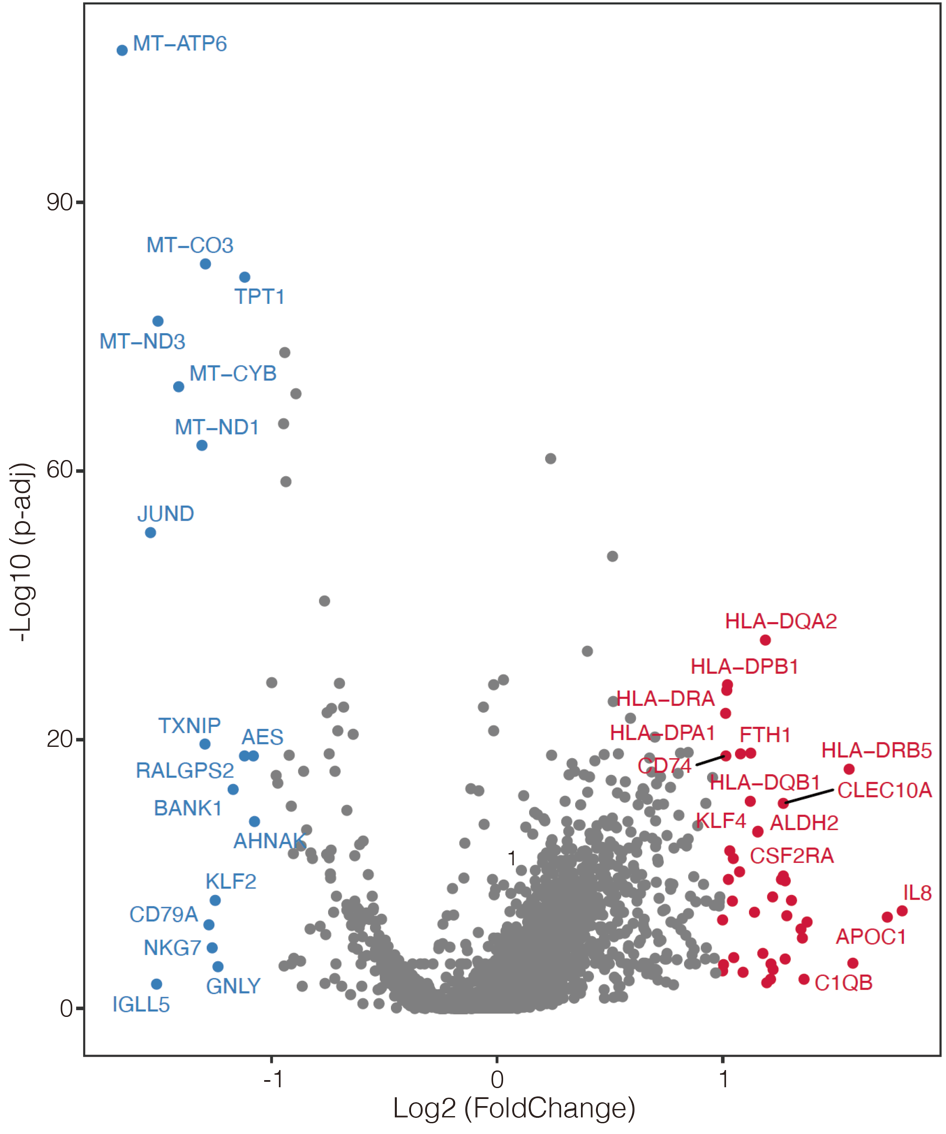


**Supplementary Figure 16.** Volcano plot showing genes in cluster Breg-C3 cluster between normal and tumor tissues. Difference between cells in two subset was plotted against log fold change of log p-value. P-value < 0.05, Two-sided unpaired limma-moderated t test; log2(fold change) >=1.4


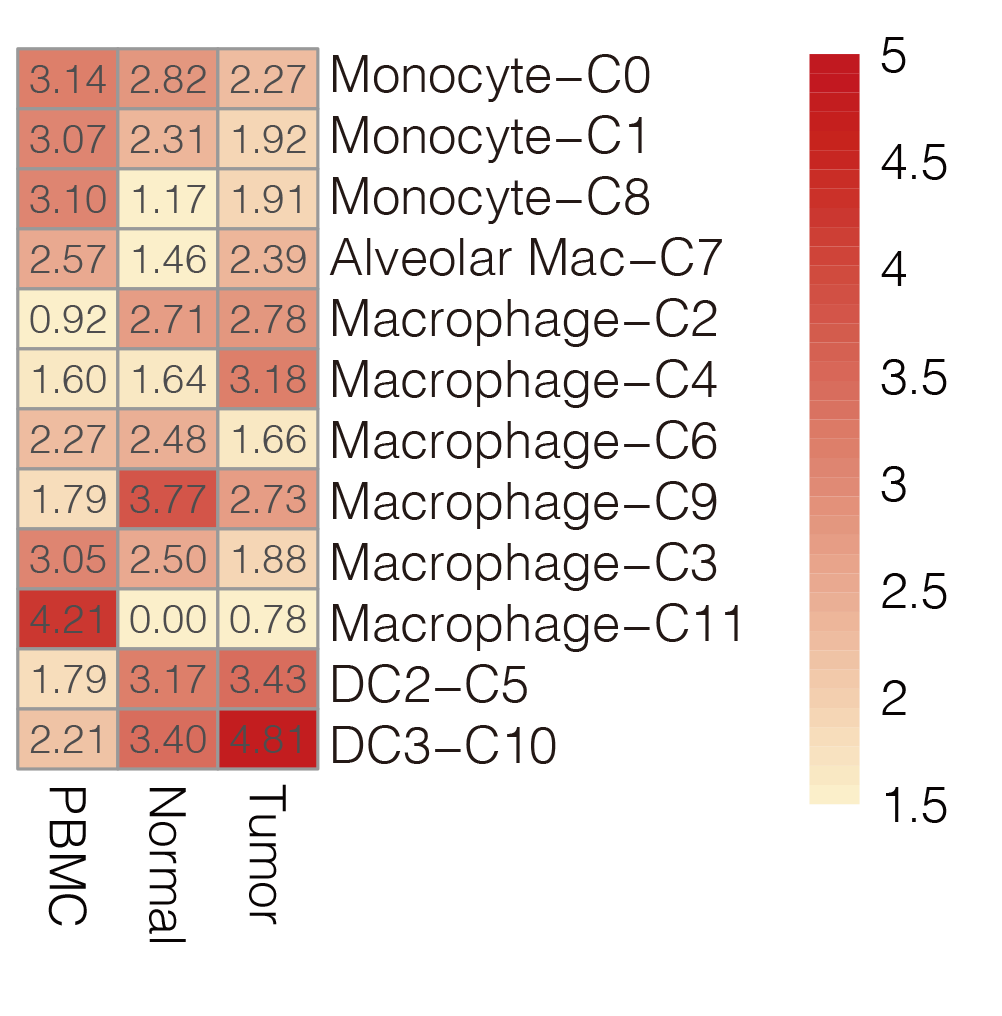


**Supplementary Figure 17.** Tissue distribution pattern of clusters of myeloid cells, based on the ratio of cell numbers (Ro/e) in each tissue. PBMC, peripheral blood mononuclear cell.


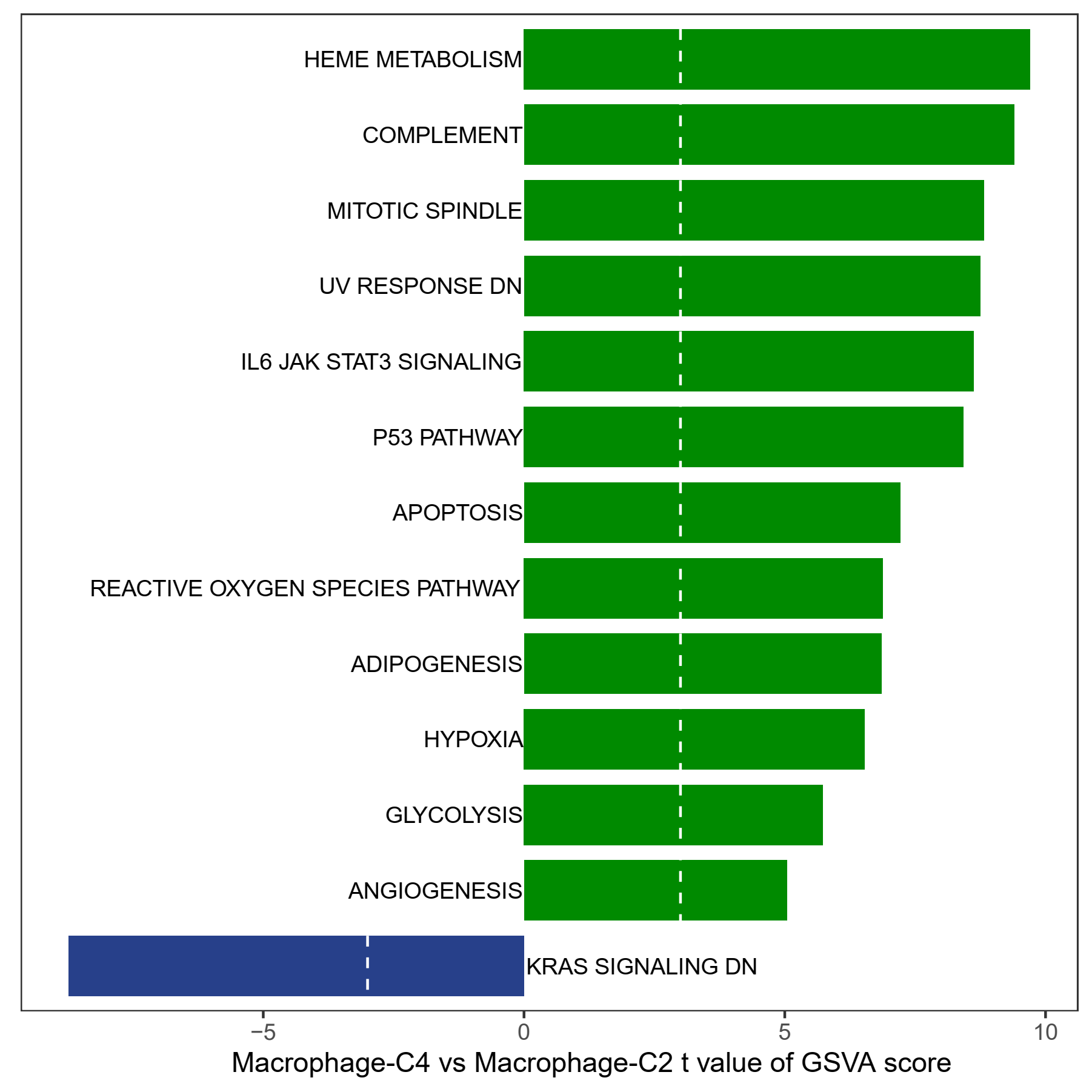


**Supplementary Figure 18.** Differences in pathway activity scores per cell between cluster Macrophage-C4 (n = 890) and Macrophage-C2 (n = 1030), based on gene set variation analysis. Shown are t values from a linear model, corrected for patient of origin. Green: macrophage-C2; Blue: macrophage-C4.


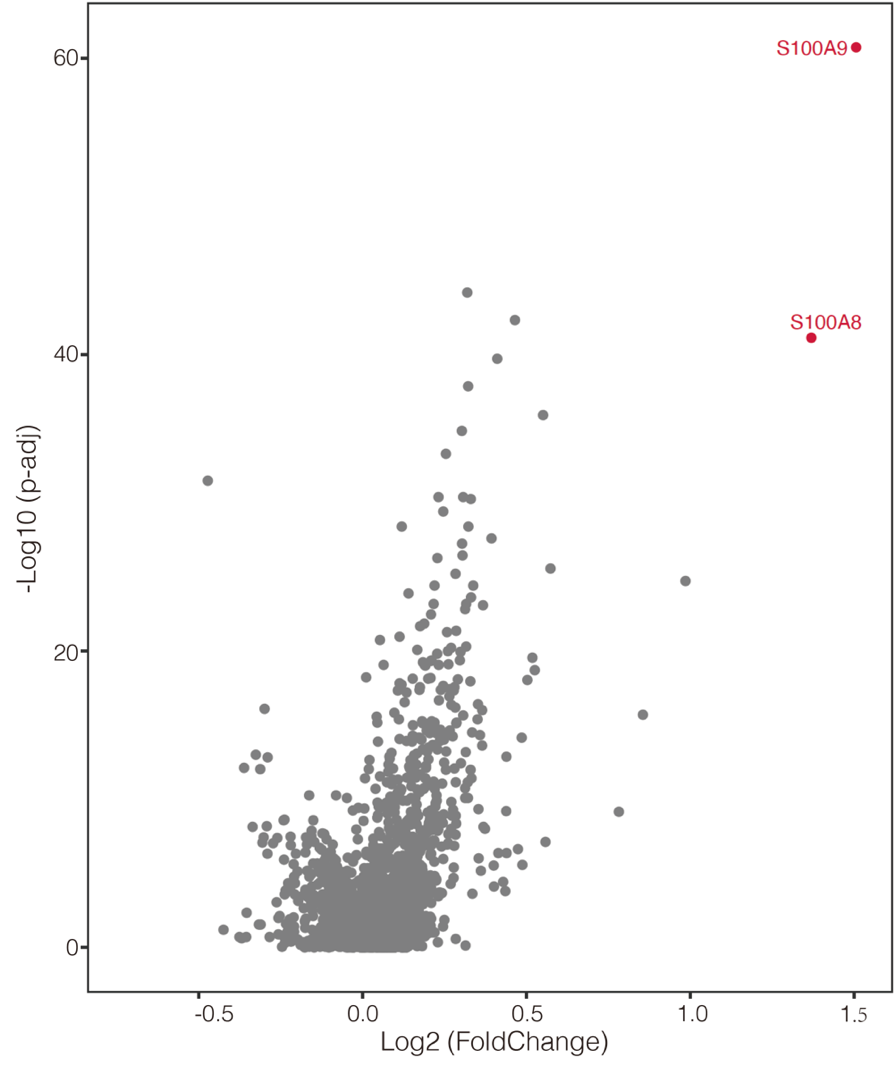


**Supplementary Figure 19.** Volcano plot showing genes in cluster Macrophage-C2 and Macrophage-C4 cells. Difference between cells in two subset was plotted against log fold change of log p-value. P-value < 0.05, Two-sided unpaired limma-moderated t test; log2(fold change) >=1.4


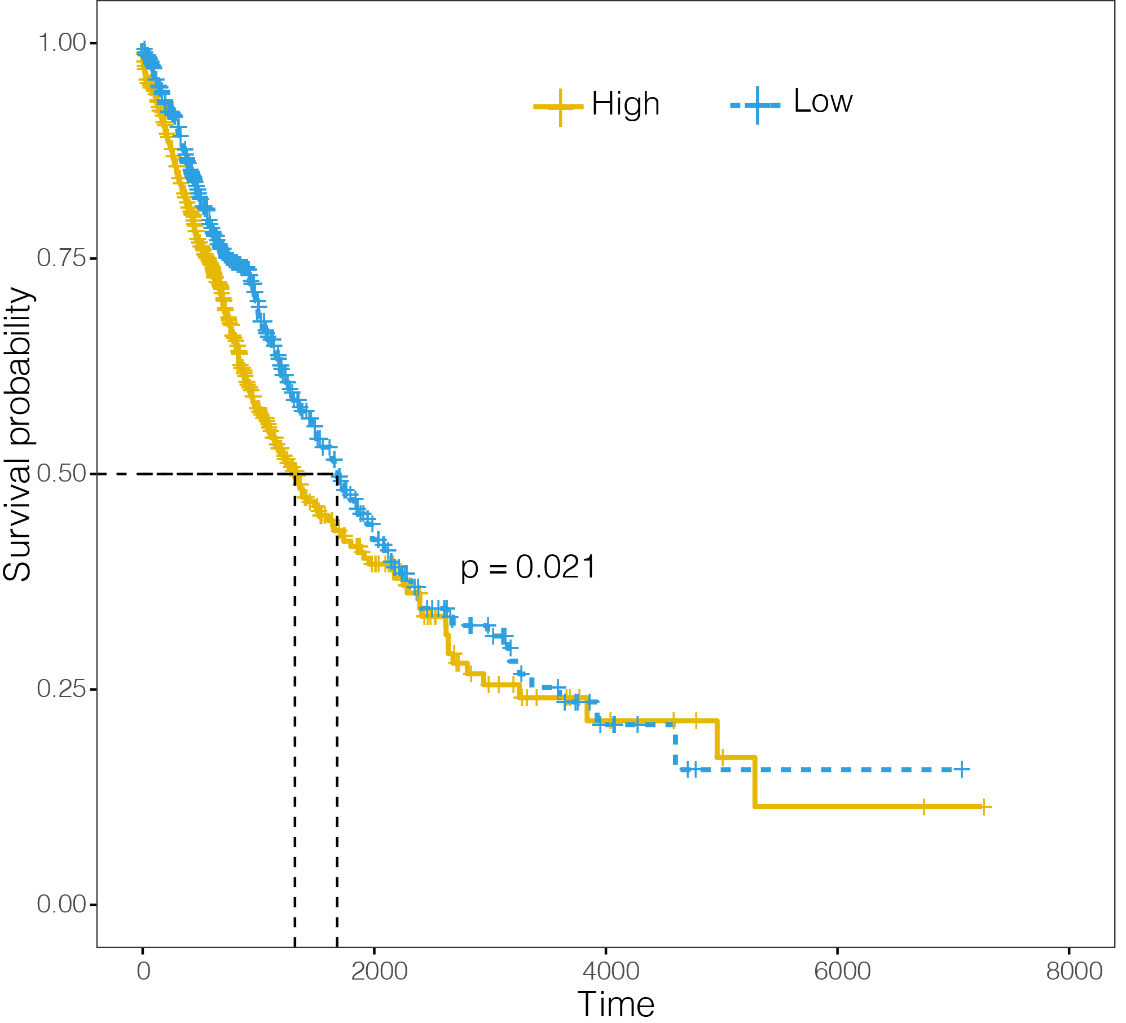


**Supplementary Figure 20.** Kaplan–Meier curves of survival of patients with NSCLC in The Cancer Genome Atlas dataset, stratified by low or high expression of BHLHE40. (Time, day)
